# Updating the Geologic Barcodes for South China: Discovery of Late Archean Banded Iron Formations in the Yangtze Craton

**DOI:** 10.1038/s41598-017-15013-4

**Published:** 2017-11-08

**Authors:** Hui Ye, Chang-Zhi Wu, Tao Yang, M. Santosh, Xi-Zhu Yao, Bing-Fei Gao, Xiao-Lei Wang, Weiqiang Li

**Affiliations:** 1 State key laboratory for Mineral Deposits Research, School of Earth Sciences and Engineering, Nanjing University, Nanjing, 210093 China; 2 School of Earth Sciences and Resources, China University of Geosciences, Beijing, 100083 China; 3 Department of Earth Sciences, University of Adelaide, Adelaide, SA 5005 Australia

## Abstract

Banded iron formations (BIFs) in Archean cratons provide important “geologic barcodes” for the global correlation of Precambrian sedimentary records. Here we report the first finding of late Archean BIFs from the Yangtze Craton, one of largest Precambrian blocks in East Asia with an evolutionary history of over 3.3 Ga. The Yingshan iron deposit at the northeastern margin of the Yangtze Craton, displays typical features of BIF, including: (i) alternating Si-rich and Fe-rich bands at sub-mm to meter scales; (ii) high SiO_2_ + Fe_2_O_3total_ contents (average 90.6 wt.%) and Fe/Ti ratios (average 489); (iii) relative enrichment of heavy rare earth elements and positive Eu anomalies (average 1.42); (iv) and sedimentary Fe isotope compositions (δ^56^Fe_IRMM-014_ as low as −0.36‰). The depositional age of the BIF is constrained at ~2464 ± 24 Ma based on U-Pb dating of zircon grains from a migmatite sample of a volcanic protolith that conformably overlied the Yingshan BIF. The BIF was intruded by Neoproterozoic (805.9 ± 4.7 Ma) granitoids that are unique in the Yangtze Craton but absent in the North China Craton to the north. The discovery of the Yingshan BIF provides new constraints for the tectonic evolution of the Yangtze Craton and has important implications in the reconstruction of Pre-Nuna/Columbia supercontinent configurations.

## Introduction

**Figure 1:**
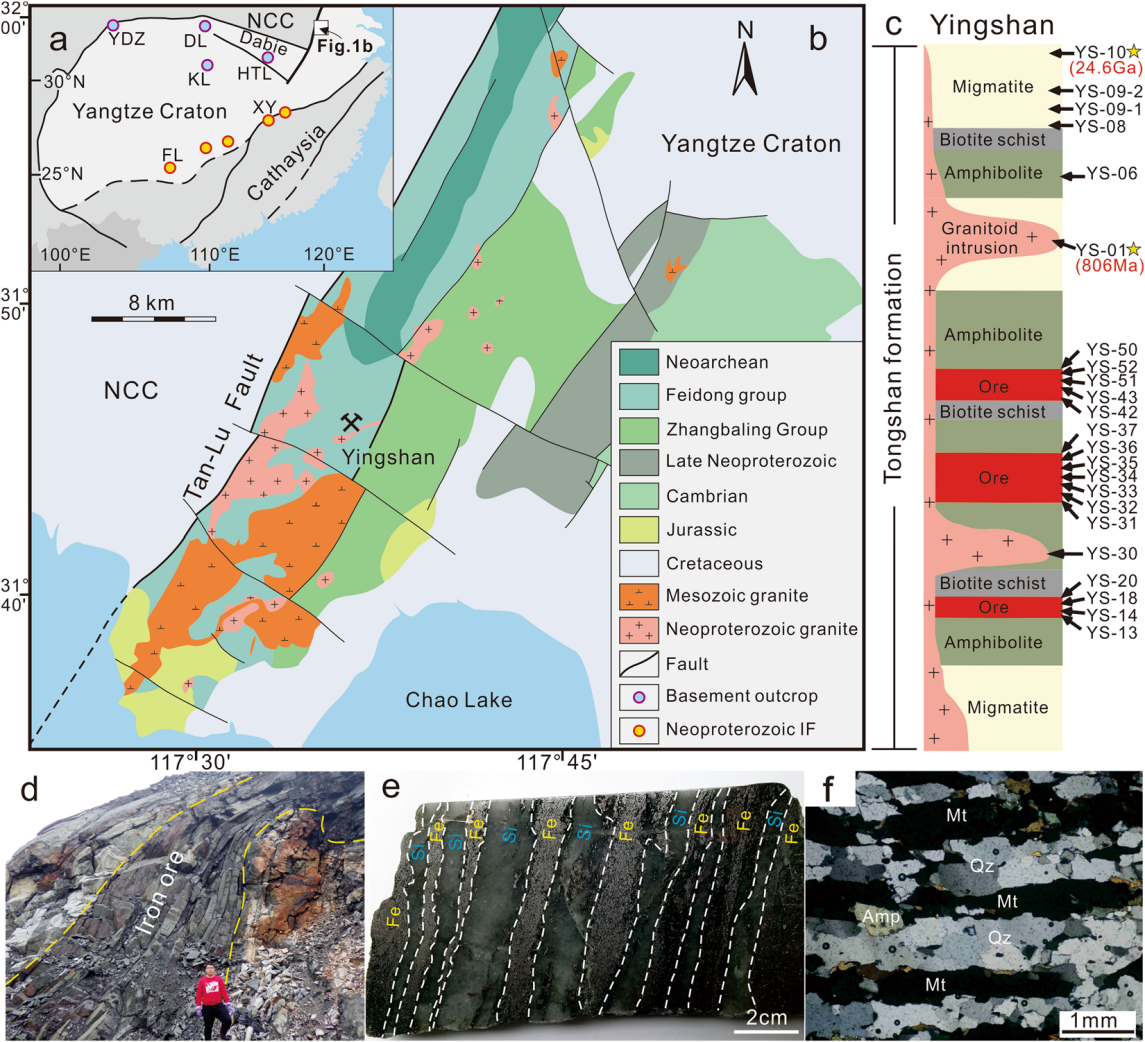
Location (**a**) and geological map (**b**) of the Yingshan iron deposit. Stratigraphic column of the Yingshan iron deposit (**c**) showing sampling point (arrow) and zircon U-Pb age (star). Representative photos showing macroband (**d**), mesoband (**e**), and microband (**f**) textures of the iron ores from Yingshan, that are similar to those of typical BIF bands elsewhere in the world such as the Dales Gorge Member, Hamersley basin. Acronyms in Fig. 1a: NCC-North China Craton; YDZ-Yudongzi group; DL-Douling complex; KL-Kongling complex; HTL-Huangtuling granulites; XY-Xinyu iron formation (Neoproterozoic); FL-Fulu iron formation (Neoproterozoic). The geological maps were generated using CorelDRAW Graphcs Suite 2017, http://www.coreldraw.com/cn/free-trials/?topNav=cn.

The Archean-Paleoproterozoic boundary was a critical period in Earth’s history with a series of significant changes in atmosphere, lithosphere and hydrosphere^1,2^. The formation of BIFs also reached its climax during this period^3^. The BIFs worldwide are important repositories of the early Earth’s major environmental transitions and biological innovations^2–4^. They are considered to have formed in distinct tectonic settings. For example, the Superior-type BIFs are thought to have developed on continental shelf below storm wave base, and granular iron-formations such as the Gunflint-type BIFs developed in shallow-water, high-energy environments^3^. In contrast, the Neoproterozoic iron formations are commonly associated with continental rift-basins^5^. Records of BIFs therefore provide important constraints on the tectonic histories of Earth’s ancient crustal blocks. The Yangtze Craton (Fig. 1a) in South China is one of the largest Precambrian blocks in East Asia, with an Archean-Paleoproterozoic basement^6,7^ that dates back to 3.3 Ga^8,9^. Although a number of Neoproterozoic iron formations occur around the southeastern margin of the Yangtze Craton (Fig. 1a), so far there has been no report on Archean-Paleoproterozoic BIFs from this craton^10,11^, which is in sharp contrast with the North China Craton to the north where Archean-Paleoproterozoic BIFs are abundant^11,12^.

Whether the absence of BIFs in the Yangtze Craton is a preservational issue or it reflects the lack of a favorable tectonic environment for Archean-Paleoproterozoic BIF deposition is a crucial question, particularly in understanding the evolution of this craton and its position with respect to pre-Nuna/Columbia supercontinents^13^. Occurrence of magnetite quartzite has been mentioned from the Archean-Paleoproterozoic Feidong Group^14^ and the Archean Yudongzi group^15^ around the boundary between the North China Craton and the Yangtze Craton (Fig. 1a), but their protolith, age and tectonic affinity remain elusive. In this contribution, we place geochronological and geochemical constraints on the magnetite quartzite from the Paleoproterozoic Feidong Group^16,17^. Our data provide unequivocal evidence for ca. 2.46 Ga BIFs from the Yangtze Craton, confirming the first discovery of late Archean BIFs in this craton.

## Geological setting, samples, and analyses

The Yangtze Craton, separated from the North China Craton by the Qinling-Sulu-Dabie orogen to the north, contains a widespread Archean basement^6,7^. Much of the basement is covered by weakly metamorphosed Neoproterozoic (eg., Lengjiaxi group and Banxi group) and Phanerozoic strata, with limited Archean-Paleoproterozoic outcrops restricted to the northern part of the craton (e.g., Kongling complex >3.3 Ga; Fig. 1a)^6–8^.

The Tan-Lu fault, the largest fault system in East Asia, defines the eastern boundary between the North China Craton and the Yangtze Craton (Fig. 1a,b). The fault sinistrally offsets the Sulu-Dabie orogen by a maximum apparent displacement of ~400 km, exposing a NEE-trending belt of the basement of the Yangtze Craton, locally termed as the Zhangbaling metamorphic belt^17,18^. This belt is composed of the greenschist-facies Neoproterozoic Zhangbaling Group in the north and the amphibolite-facies Paleoproterozoic Feidong Group in the south^17^. The Feidong Group contains the Fuchashan, the Tongshan and the Qiaotouji formations from bottom to top. The Tongshan Formation is a metamorphosed sedimentary-volcanic succession and contains several thin-bedded magnetite quartzite layers that extend over 30 km (Fig. 1c).

The Yingshan iron deposit is hosted in the Tongshan Formation of the Feidong Group. The ore bodies occur as Fe-rich layers that typically extend for over 1 km with an average thickness of over 10 m and Fe grade of 27 wt.% (Supplementary Fig. S1). The ores consist of banded quartz-magnetite and garnet-amphibolite-quartz-magnetite (Supplementary Fig. S1). The layered ore bodies are inter-bedded with amphibolite, biotite schist and migmatite, and have been intruded by granitoids (Supplementary Fig. S1). The rocks locally underwent amphibolite-grade metamorphism, structural deformation and hydrothermal alteration associated with the Tan-Lu fault system (Supplementary Fig. S1) during the Triassic^17^.

Twenty-six samples were collected from the Yingshan deposit (Fig. 1c), including iron ores from three layers of orebodies, as well as the host rocks including migmatite, biotite schist and amphibolites. Bulk rocks were pulverized and analyzed for major and trace elements using XRF and ICP-MS, respectively. Iron isotope compositions of the bulk rocks were measured by solution-nebulization MC-ICP-MS after ion-exchange purification. Zircon grains were separated from a leucosome (YS-10) sample from the upper wall of the ore bodies, and a granitoid (YS-01) that intruded into the iron ore bodies (Fig. 1c). The grains were analyzed using LA-ICP-MS for U-Pb dating. Details of the results are provided in the Supplementary Information.

## Results and Discussion

### Protolith of the iron ores

In spite of the deformation and metamorphism, the iron ores from Yingshan show characteristic banded texture with alternating silica-rich and iron-rich layers. The banded textures are obvious in meter scale within the layered ore bodies (Fig. 1d), at centimeter scale in hand specimens (Fig. 1e), and at sub-millimeter scale within iron-rich bands under the microscope (Fig. 1f). These are consistent with the classic macroband, mesoband, and microband textures of typical BIFs as for example in the case of the Dales Gorge Member in the Archean-Proterozoic Hamersley basin, western Australia^19^.

**Figure 2:**
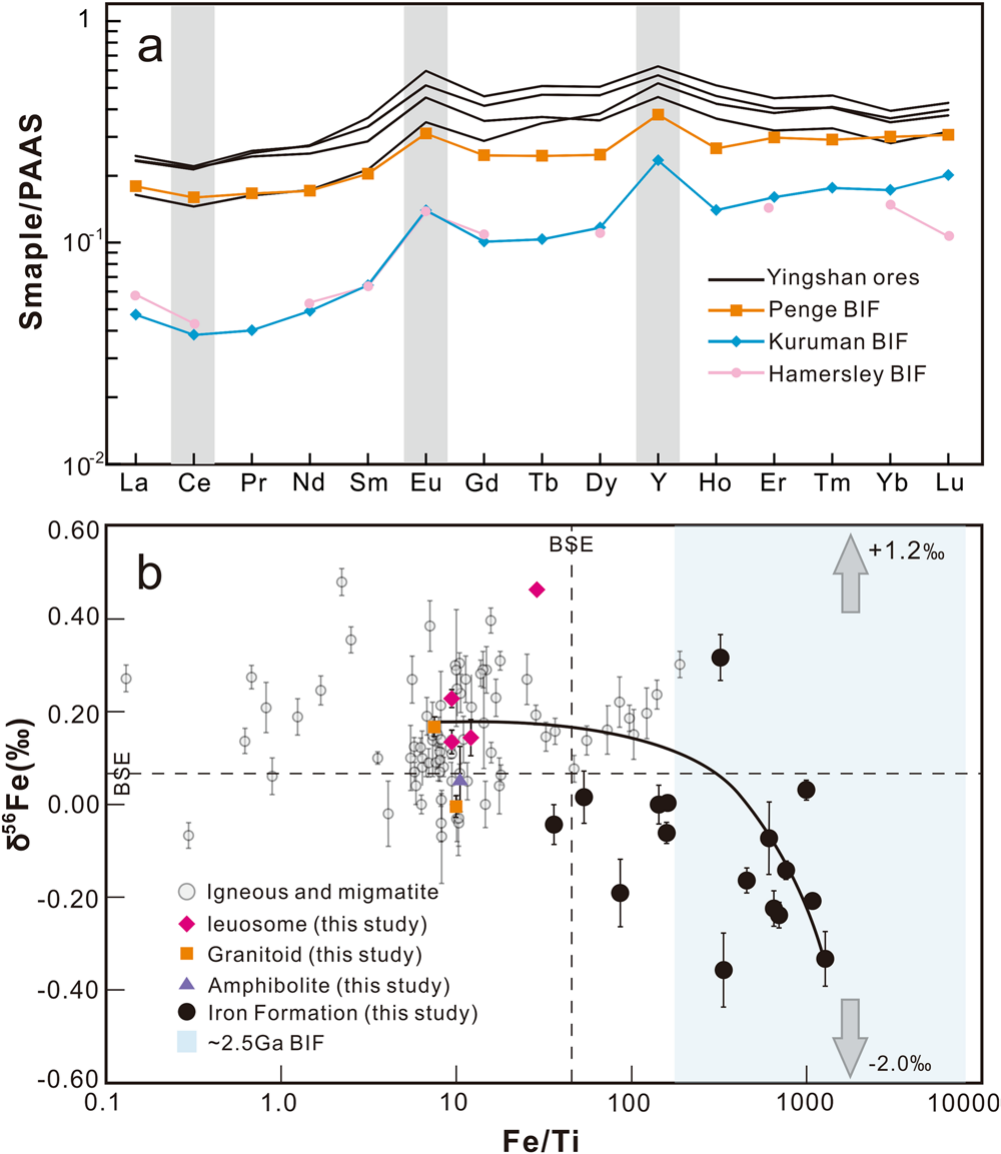
Plots of rare earth element plus yttrium (REE + Y) patterns (**a**), and δ^56^Fe (‰ relative to IRMM-014) versus Fe/Ti ratios (**b**) for samples from the Yingshan iron deposit. For comparison, the REE + Y patterns of ~2.5 Ga Hamersley, Penge and Kuruman BIF are also plotted in Fig. 2a. Data of igneous rocks (Supplementary Table S2) and banded iron formations (Supplementary Table S3) are plotted as light gray dots and light yellow shaded rectangles, respectively. The solid curve denotes the mixing trend between YS-01 (granitoid) and YS-43 (banded ore).

Major elements of the banded iron ores (Supplementary Table S1) are dominated by SiO_2_ and Fe_2_O_3Total_ (79.4–95.8 wt%), a feature characteristic for oceanic chemical deposits, and consistent with the average composition of BIFs as summarized from 214 BIFs worldwide^3^. The variable contents of Al_2_O_3_ (1.66–9.81 wt%) and TiO_2_ (0.04–0.86 wt%) likely reflect syndepositional volcanic inputs. Additionally, ore samples with low Al_2_O_3_ and TiO_2_ contents display rare earth element plus Y (REE + Y) patterns of relative enrichment in heavy REE (average HREE/LREE* = 2.28) and positive Eu anomalies (average = 1.42) (Supplementary Table S4). The REE + Y patterns of the iron ores are similar to those of the classic BIFs formed during the Archean-Proterozoic transition (Fig. 2a).

Iron isotope analyses for all the 23 samples (Fig. 2b, Supplementary Table S1) revealed a variation of 0.83‰ in δ^56^Fe. The wall-rock samples, including leucosome of migmatite, amphibolite and granitoid intrusion, have positive δ^56^Fe values (0.00–0.47‰). In contrast, ore samples are generally enriched in light Fe isotopes, with δ^56^Fe ranging from −0.36‰ to 0.04‰ with an exception of one sample that has δ^56^Fe of +0.32‰ (Fig. 2b). Magmatic rocks in general possess a homogeneous Fe isotope composition^20,21^, except for highly evolved (SiO_2_ > 70 wt%) granitoids and leucosome of migmatites that have high δ^56^Fe values^22,23^. The variable and low δ^56^Fe values of the iron ores therefore exclude an igneous parentage, and instead reflect Fe isotope variation in the protolith. The complexities in bulk rock Fe isotope compositions caused by mixing with detrital components as well as metamorphism and hydrothermal alteration can be assessed using a plot of δ^56^Fe versus Fe/Ti. This approach has proven to be effective in resolving the protolith of the earliest BIF from the highly metamorphosed 3.8 billion-year-old rocks of Isua, Greenland^24^ (Fig. 2b). There is a general negative correlation between the Fe/Ti atomic ratio and δ^56^Fe values for the iron ores, reflecting mixing between an igneous Fe end member and an end member that is characterized by a high (>1200) Fe/Ti ratio and low δ^56^Fe (Fig. 2b). Low δ^56^Fe values are one of the hallmarks of BIFs that are absent in other bulk geological samples, and are considered to reflect redox processes during deposition of Fe in the water column and subsequent diagenesis in soft sediments^4,25^.

### Depositional age and tectonic affinity of the Yingshan BIF

**Figure 3:**
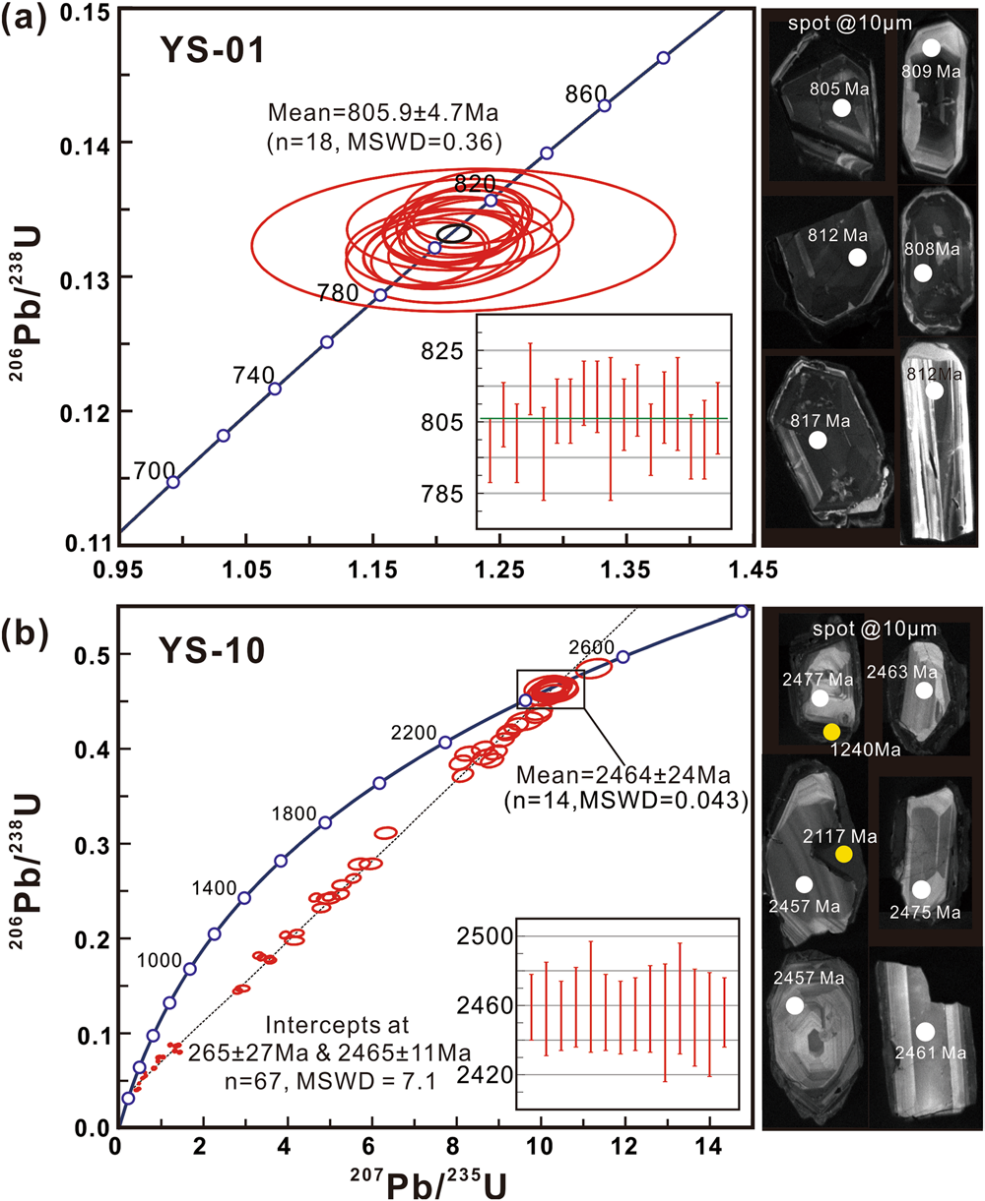
Zircon U-Pb Concordia and representative cathodoluminesence (CL) images for leucosome (YS-01) and granitoid intrusion (YS-10), respectively.

Multiple lines of evidence from texture, chemical compositions and Fe isotope compositions presented above collectively indicate that the protolith for the iron ores from the Yingshan deposit was a banded iron formation, which is referred to as the Yingshan BIF hereafter. The depositional age of the Yingshan BIF is constrained by U-Pb geochronology of zircon grains from the leucosome (YS-10) of a migmatite. The protolith of the migmatite is thought to be a volcanic rock interbedded within the Tongshan volcano-sedimentary sequence and conformably overlying the Yingshan BIF mineralization. Association with volanic rocks is a common feature for Archean BIFs^2,3^, particularly for Algoma-type BIFs^2^, and the age of zircons from the volcanic layers interbedded in BIFs have been widely used to constrain the depositional ages of the BIFs^26^. The zircons from the migmatite leucosome are euhedral with a size of 40–170 μm, and cathodoluminescence (CL) imaging reveals common core-rim textures (Fig. 3). The zircon cores show bright oscillatory CL zoning, and have high Th/U ratio (Th/U = 0.45–1.16), as well as HREE-enriched patterns with positive Ce and Sm anomalies (Fig. 3; Supplementary Fig. S2), all of which are typical of magmatic zircons^27^. The rims of the zircon in contrast are dark-gray in CL, have low Th/U values (Th/U = 0.04–0.07) and flat REE patterns without significant Ce and Sm anomalies Fig. 3; Supplementary Fig. S2), suggesting a metamorphic origin^28^. The concordant U-Pb ages for the magmatic zircon cores tightly cluster at 2464 ± 24 Ma (^207^Pb/^206^Pb age, n = 14, MSWD = 0.04). The tight distribution of U-Pb ages as well as the irregular shapes of the zircon cores supports the idea that the protolith of the migmatite is a volcanic rock rather than a detrital sediment^27,28^. Except for an inherited zircon with a concordant^207^Pb/^206^Pb age of 2544 Ma (Fig. 3, Supplementary Table S5), all the U-Pb analyses of cores and rims of zircon grains from YS-10 define a Discordia with an upper intercept age of 2465 ± 11 Ma and a lower intercept age of 265 ± 27 Ma (n = 67, MSWD = 7.1; Fig. 3). The upper intercept age of the Discordia is consistent with the concordant age (2464 ± 24 Ma) from the zircon cores, representing the age of the volcanic protolith of the migmatite, whereas the lower intercept age reflects an early Mesozoic thermal event that produced the metamorphic rims of the zircon grains. Because of the conformable relationship between the volcanic protolith of the migmatite and the iron formation (Fig. 1c; Supplementary Fig. S1), the Yingshan BIF is interpreted to be approximately coeval with the volcanic protolith of the migmatite corresponding to late Archean-early Paleoproterozoic (2464 ± 24 Ma).

**Figure 4:**
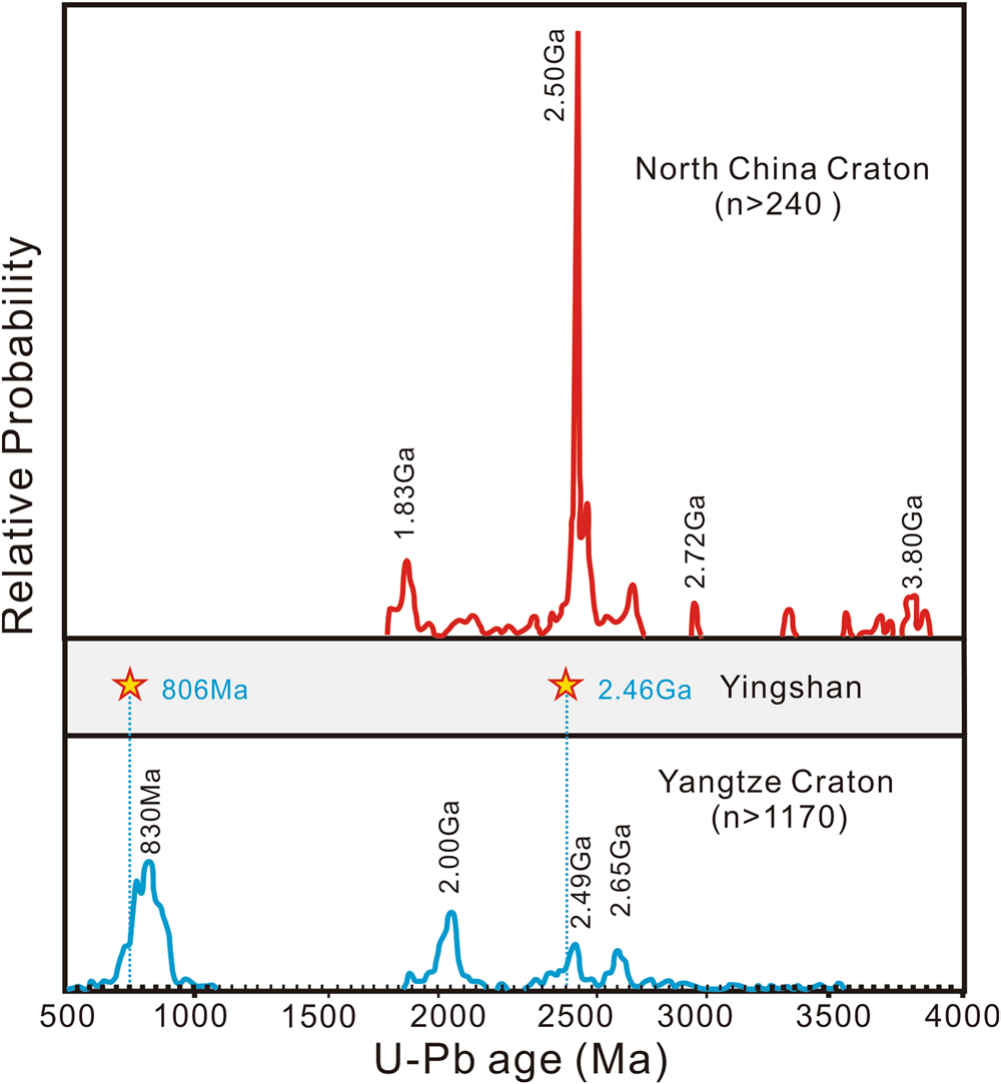
Comparison of Precambrian detrital zircon U-Pb age spectra between the Yangtze Craton and the North China Craton (modified after^30^). The U-Pb ages of zircons from the Yingshan iron deposit in this study are represented by stars.

Because the Yingshan iron deposit is located within a major fault zone between the Yangtze Craton and the North China Craton, it is crucial to ascertain the tectonic setting of the BIF protolith. The Yangtze Craton and the North China Craton did not collide until the Triassic^17^, and the two cratons have distinct Precambrian evolution histories (Fig. 4). The North China Craton is characterized by widespread Archean magmatism that peaked at ~2.5 Ga^29,30^ and very minor Neoproterozoic magmatic activities in the form of mafic dykes^31^. In contrast, the Yangtze Craton is characterized by widespread Neoproterozoic magmatism^32,33^ and ca. 2.0 Ga magmatic events and metamorphism^34^. The Yingshan iron deposit was intruded by a granitoid pluton (Fig. 1c). Zircon grains from the granitoid (YS-01) are dark gray to gray in color, euhedral to subhedral with a size of 25–150 μm and aspect ratio of 0.5–6.0 (Fig. 3). These zircons show bright oscillatory zoning in CL imaging (Fig. 3) revealing their magmatic origin. U-Pb ages of these magmatic zircons from YS-01 are concordant and yield a weighted mean^206^Pb/^238^U age of 805.9 ± 4.7 Ma (n = 18, MSWD = 0.36). The age of the granitoid intrusion is consistent with the magmatism along the northern margin and elsewhere within the Yangtze Craton but is conspicuously absent in the southern margin of the North China Craton. The age data suggest that the Yingshan iron ore bodies were intruded by Neoproterozoic (805.9 ± 4.7 Ma) granitoid prior to the collision between the Yangtze Craton and the North China Craton, thus confirming that the Yingshan BIF belongs to the Yangtze Craton (Fig. 4).

## Geological Implications

Based on the geological, geochemical and geochronological evidence presented above, the Yingshan iron deposit is identified as a Neoarchean-Paleoproterozoic banded iron formation and provides a first case of BIF mineralization of such age in the Yangtze Craton. The discovery of the Yingshan BIF indicates that the northern part of the Yangtze Craton was in a shallow marine to continental shelf environment during the late Archean. Occurrence of the late Archean BIF on the northern margin of the Yangtze Craton is in contrast with the linear distribution of Neoproterozoic BIFs in the southern margin of the Yangtze Craton that are indicative of rifting during the Neoproterozoic (Fig. 1a). Such contrast in BIF occurrence seems to reflect a tectonic polarity for the Yangtze Craton, which is interestingly perpendicular to the northern boundary with the North China Craton and the southern boundary with the Cathaysia Block. This feature might have important implications on the patterns of amalgamation and breakup of continents and direction of craton drifting, at least for the case of the Yangtze Craton. The discovery of the Yingshan BIF also places further constraints for understanding the evolution of the Yangtze Craton during the late Archean. The depositional age (~2.46 Ga) of the Yingshan BIF is consistent with the 2.40 ~ 2.55 Ga peak derived from detrital zircon age spectra of the Yangtze Craton^30^. This is also concordant with the age (2493 ± 19 Ma) of TTG from the basement of the Neoproterozoic Zhangbaling Group, and the age (~2.5 Ga) of the protolith of sheared dioritic-granitic rocks from the Douling complex of southern Qinling orogeny^35^. Therefore, it is evident that the deposition of the Yingshan BIF was accompanied by a tectono-magmatic event in the Yangtze Craton which also correlates with the global peak in magmatism during the Archean to Paleoproterozoic transition^36^ (ca. 2.45Ga).

**Figure 5:**
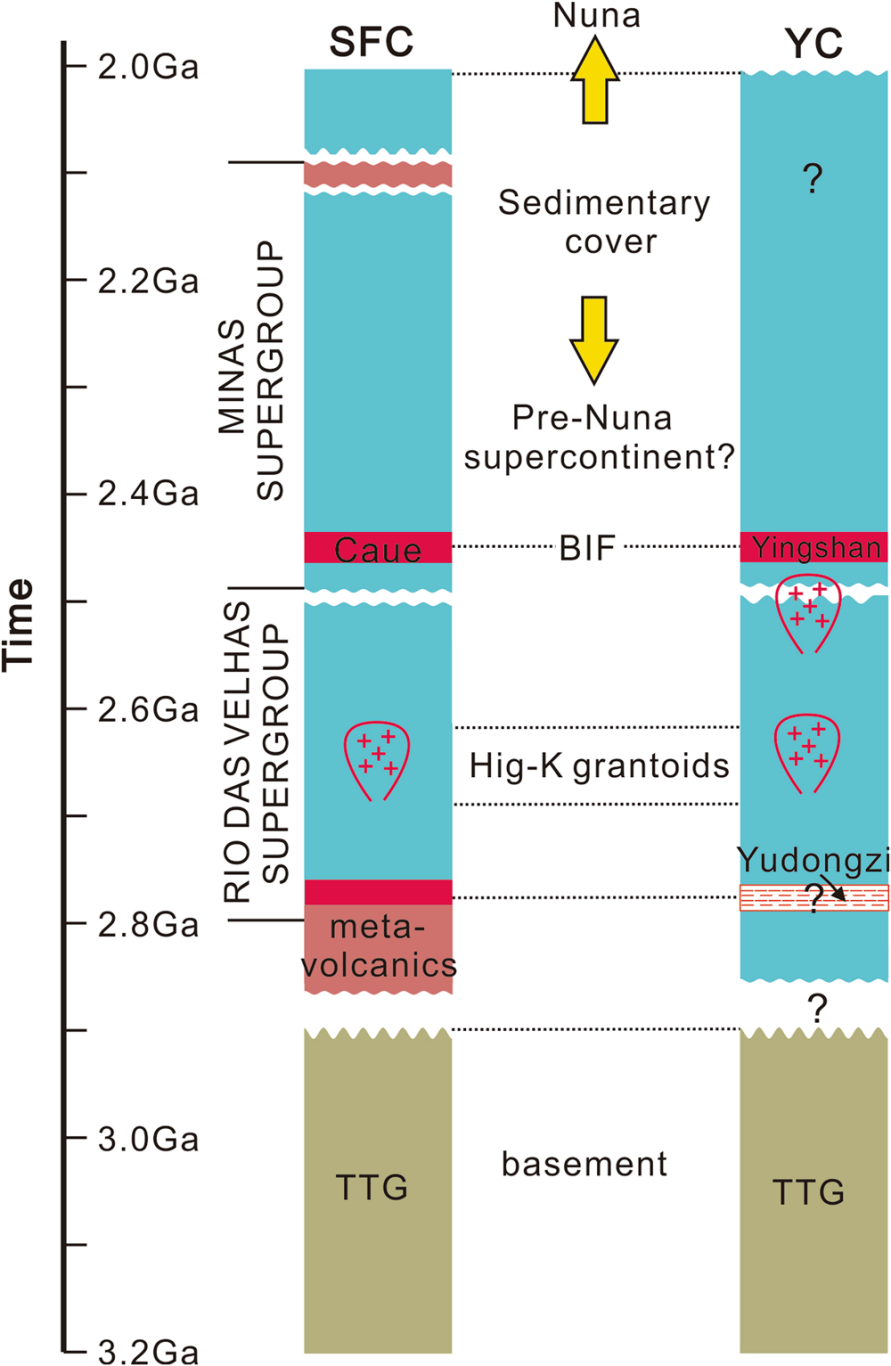
Comparison of stratigraphic-tectonic histories between the Southern Sao Francisco Craton (SF) and the Yangtze Craton (YC).

Banded iron formations are considered as important “geologic barcodes” for the reconstruction of supercontinents in Earth’s deep time^37–39^. For example, the similarity between Archean-Paleoproterozoic BIF records in the Pilbara Craton in Western Australia and the Kaapvaal Craton in South Africa lays the foundation for the idea that these two cratons were once part of a supercraton (the Vaalbara) or the same sedimentary basin^40^. We note remarkable similarity in the tectonic-sedimentary history between the Yangtze Craton and the Sao Francisco Craton of South America, including a 3.2–2.9 Ga basement of TTG, ~2.7 Ga high-K granitoid magmatic episode, ~2.5 Ga unconformity, ~2.46 Ga BIF (Yingshan and Caue BIFs), 2.4–2.0 Ga sedimentary cover (Fig. 5). Discovery of the Yingshan BIF, therefore, provides additional geological constraints from the Yangtze Craton for further evaluation of the Pre-Nuna/Columbia supercontinents^39,41^.

## Methods

### Sample preparation

All 23 wall/ore samples collected from the Yingshan iron deposit were cut to remove weathered surfaces. These relatively fresh samples were cleaned, dried and pulverized for compositional analysis. Zircon grains from the leucosome (YS-10) and granitoid (YS-01) sample were separated using standard crushing, heavy liquid, magnetite separation, and hand-picking techniques, then mounted in epoxy resin and polished for U-Pb isotope analysis. All analyses were done in the state key laboratory for mineral deposits research, Nanjing University.

### Whole-rock major element and trace element analyses

Whole-rock major elements were analyzed using an ARL9800XP and X-ray fluorescence spectrometer (XRF), which gives the analytical precision better than 2% for all major elements. Whole-rock REEs abundances were measured using a Finnigan Element II ICP-MS and gives precision better than 10% for most REEs. Major element and selected trace element (REE + Y) results are provided in Supplementary Tables S1 and S4.

### Whole-rock iron isotope analysis

Approximately 10 to 150 mg bulk-rock powder for each sample was digested in a 2:1:1 mixture of concentrated HCl-HNO_3_-HF in 7 mL Teflon beaker on hot-plate at ~130 °C for 2 days. After evaporation, the samples were completely dissolved in a 3:1 mixture of concentrated HCl-HNO_3_ and dried again. The fully dissolved samples were converted to chloride form by repeated redissolution in 1 mL concentrated HCl and subsequent evaporation to dryness. The samples were finally dissolved in 5 mL 7 M HCl and stored in a Teflon beaker as sample stock solution. Based on measured Fe concentrations, an aliquot of the sample stock solution that contained 100 μg Fe was extracted and evaporation to dryness and then dissolved in 100 μL 7 M HCl for chemical purification.

Iron was separated from matrix elements by anion exchange chromatography^42^ using 0.2 mL Bio-Rad AG MP-1 resin in a custom-made shrinkable Teflon column (4mm ID, 26mm height). Before anion exchange, the resin was cleaned with 1000 μL 2% (volume ratio) HNO_3_ and 1000 uL Milli-Q H_2_O, then conditioned with 2000 μL 7 M HCl. After loading 100 μL sample solution in 7 M HCl onto the resin, the matrix elements were eluted off the column using 3 mL of 7 M HCl in 0.5 mL increments. Iron was subsequently eluted from the resin using 3 mL of 2% HNO_3_. The Fe cut was evaporated to dryness, redissolved in 100 μL sample in 7 M HCl, and was purified for a second time by repeating the anion exchange procedure as described above. Purified Fe was dried and treated with three drops of 30% H_2_O_2_ and 2 mL concentrated HNO_3_ to decompose organic matters. Then Fe was dissolved in 4 mL 2% HNO_3_ and ready for mass spectrometry analysis. Recovery of Fe for the column procedure was rountinely monitored for each sample by measuring the Fe contents in solutions before and after the ion exchange chromatography using photo spectroscopy (the Ferrozine method), and the Fe recovery was >95%.

Iron isotope ratios were measured using a Thermo Fisher Scientific Neptune Plus MC-ICP-MS at State Key Laboratory for Mineral Deposit Research, Nanjing University. The instrument was running at “wet-plasma” mode using a 100 μL/min self-aspirating nebulizer tip and a glass spray chamber. Molecular interferences of ^40^Ar^14^N^+^ and ^40^Ar^16^O^+^ on ^54^Fe^+^ and ^56^Fe^+^ were fully resolved using high mass resolution setting of the instrument. Isobaric interference of ^54^Cr^+^ on ^54^Fe^+^ was monitored by simultaneous measurement of ^53^Cr^+^ signals and was corrected offline. Instrument sensitivity was 4–6 V/ppm on ^56^Fe^+^ with the instrument setting. A standard-sample-standard bracketing routine was applied for Fe isotope ratio measurement, and samples were diluted to 2 ± 0.2 ppm to match the concentration of an in-house standard that was constant at 2.0 ppm. A 40 s on-peak acid blank was measured before each analysis. Each Fe isotope ratio measurement consisted of fifty 4-s integrations, and the typical internal precision (2standard error or 2SE) was better than ±0.03‰ for ^56^Fe/^54^Fe and ±0.05‰ for ^57^Fe/^54^Fe. The long-term external reproducibility (2 standard deviation or 2 SD) of Fe isotope analysis is better than ±0.06‰ in ^56^Fe/^54^Fe and ±0.16‰ in ^57^Fe/^54^Fe over six months, based on repeat analysis of multiple Fe isotope standard solutions against in-house stock solutions.

Iron isotope compositions are reported as δ^56^Fe relative to the international standard of IRMM-014:

$${\rm{\delta }}{}^{56}{\rm{F}}{{\rm{e}}}_{{\rm{sample}}}=[{({}^{56}{\rm{F}}{\rm{e}}/{}^{54}{\rm{F}}{\rm{e}})}_{{\rm{sample}}}/{({}^{56}{\rm{F}}{\rm{e}}/{}^{54}{\rm{F}}{\rm{e}})}_{{\rm{IRMM}}\mbox{--}014}-1]\times 1000[\textperthousand ]$$

Accuracy of Fe isotope measurements was confirmed by repeated measurements of reference samples and geostandards that were treated as unknowns with the rhyolite samples. δ^56^Fe of two ultrapure Fe solutions from University of Wisconsin-Madison, J-M Fe and HPS Fe, are 0.37 ± 0.06‰ (n = 10, 2 SD) and 0.58 ± 0.06‰ (n = 7, 2 SD), respectively, which are in excellent agreements with the recommended values^22^. In addition, the measured Fe isotope compositions of the international whole-rock standards, DNC-1a (δ^56^Fe = 0.02 ± 0.06‰, n = 3), BCR-2 (δ^56^Fe = 0.11 ± 0.08‰, n = 9), BHVO-2 (δ^56^Fe = 0.13 ± 0.05‰, n = 9), BIR-1a (δ^56^Fe = 0.08 ± 0.06‰, n = 3) and DTS-2b (δ^56^Fe = 0.06 ± 0.08‰, n = 3), are all consistent with the recommended values^43,44^ within analytical uncertainties. For igneous rocks investigated in this study, each sample was measured at least three times and analytical uncertainties of Fe isotope ratios were given as 2 SD.

All 23 samples except for YS-52 in this study were analyzed at least two times. Iron isotope results relative to IRMM-014 are provided in Supplementary Table S1.

### Zircons U-Pb dating and trace element

All grains were imaged using a Mono CL4 Cathode-Luminescence (Gatan, USA) detector on a Zeiss Scanning Electron Microscope (Supra55, Germany). These CL images were used to define the shape and internal structures of the zircons, where suitable locations for laser spot analysis were chosen from (Fig. 3). U-Pb isotope and trace element measurements (Supplementary Tables S5 and S6) were conducted using a GeolasPro193nm ArF Excimer laser ablation system combined with an Element XR high resolution inductively coupled plasma mass-spectrometer (ThermoFisher, USA). Laser ablation was conducted in a helium atmosphere, running with an energy density of 6 J/cm^2^, a pulse repetition rate of 8 Hz, and a spot size of 10 µm. Each time-resolved laser ablation analysis took about 90 s, including 30 s of gas blank measurement (i.e. on-peak zeros), followed by 40 s of laser ablation and 20 s of washout time to allow the signals to drop back to background levels. Zircon standards 91500^45^ and GJ-1^46^ were used for calibration and data quality control. Raw data from mass spectrometer were reduced using a Glitter (ver 4.0) software and the U-Pb ages were calculated using Isoplot® (ver 4.15). Common-Pb corrections were carried out prior to U-Pb age calculation using a well established routine of ComPbCorr#3–15 by Andersen (2002)^47^. The U-Pb isotope and trace element composition of zircons is provided in Supplementary Tables S5 and S6.

### Statement of informed consent

Hui Ye appears in Fig. 1d as a scale bar of the orebody and he grants permission on his appearance in this figure.

## Electronic supplementary material

Supplementary Information
