# Supplementary material for: Updating the Geologic Barcodes for South China: Discovery of Late Archean Banded Iron Formations in the Yangtze Craton
Source: Sci Rep. 2017 Nov 8;7:15082. doi: 10.1038/s41598-017-15013-4 (PMC5678179; doi:10.1038/s41598-017-15013-4)
Supplement: Supplementary file 1 — Supplementary Information [file 41598_2017_15013_MOESM1_ESM.doc]

**Supplementary Material for:**

# Updating the Geologic Barcodes for South China: Discovery of Late Archean Banded Iron Formations in the Yangtze Craton

Hui Ye ^1^, Chang-Zhi Wu^1*^, Tao Yang ^1^, M. Santosh ^2,3^, Xi-Zhu Yao ^1^, Bing-Fei Gao^1^, Xiao-Lei Wang^1^, Weiqiang Li^1*^

^1^State key laboratory for Mineral Deposits Research, School of Earth Sciences and Engineering, Nanjing University, 210093, China

^2^School of Earth Sciences and Resources, China University of Geosciences, Beijing 100083, China

^3^Department of Earth Sciences, University of Adelaide, Adelaide SA 5005 Australia

## Data sources for Fig. 5.

The strata and magmatic sequences of Southern Sao Francisco (SF) and Yangtze Craton (YC) are derived from the following sources.


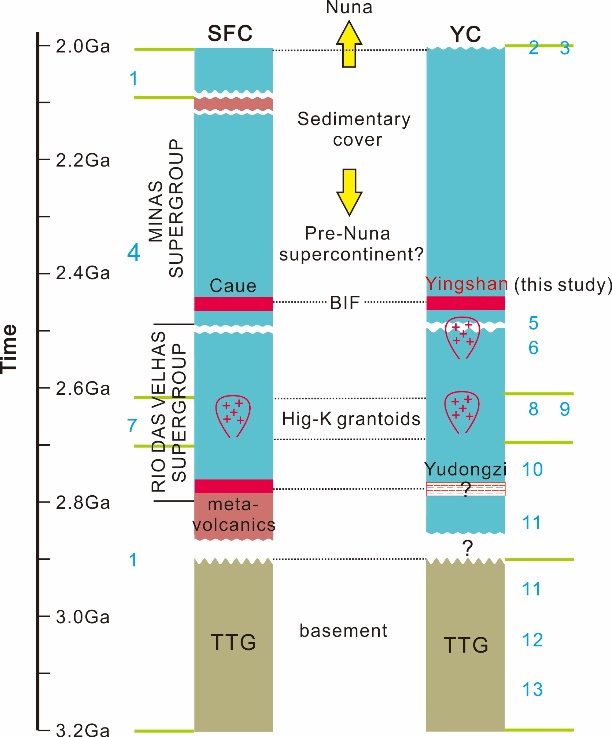


## Figures of Supplementary Material

**
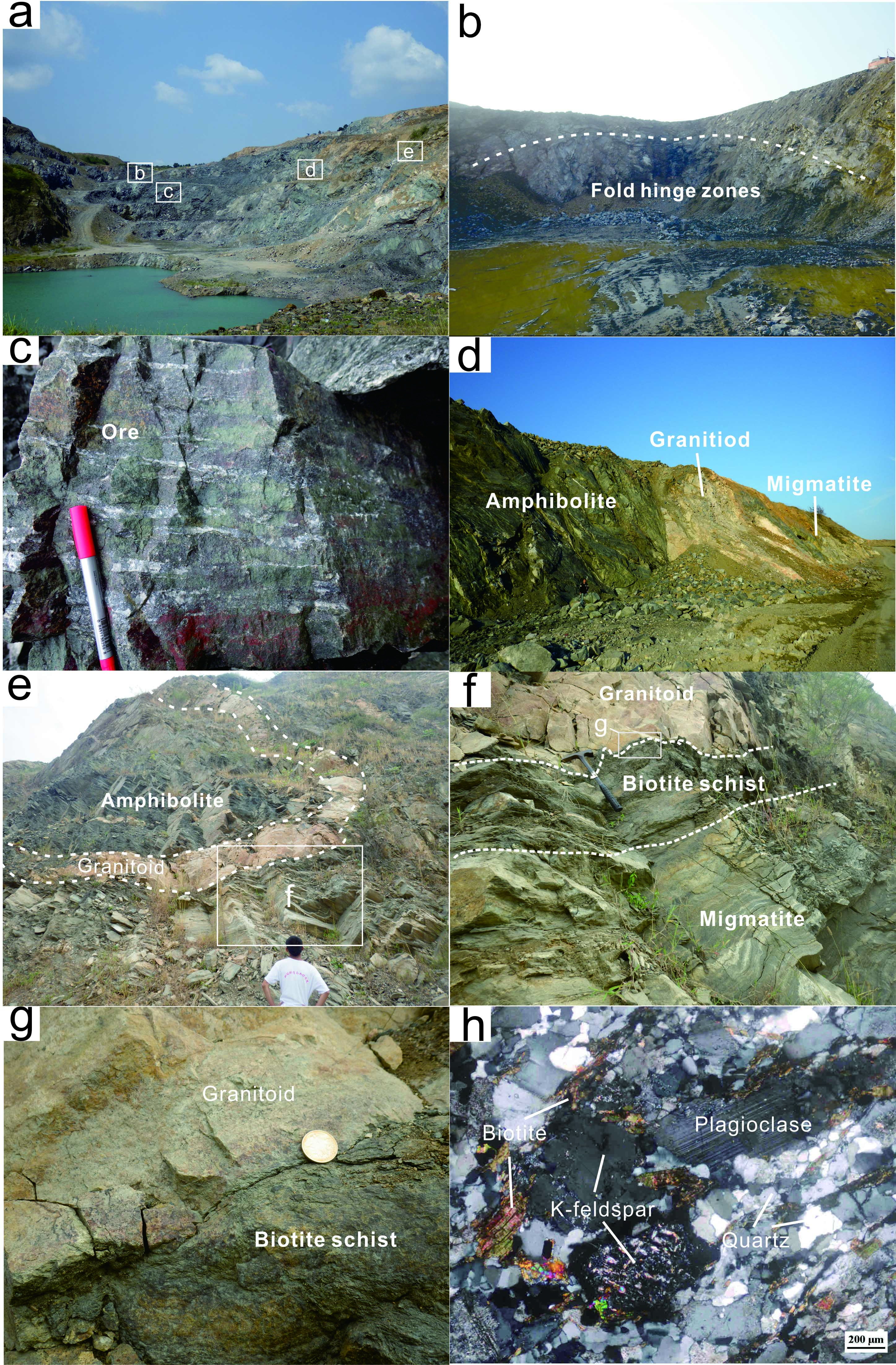
**

**Figure S1**. Field photos and photomicrographs from the Yingshan deposit, northeast margin of Yangtze Craton, South China. a) General view of the Yingshan iron deposit and contact relationship among ore body, amphibolite and migmatite; b) Thickened iron orebody within a fold; c) Banded iron ore with magnetite-rich and quartz-rich layers; d) The contact relationship among granitoid intrusion, amphibolite and migmatite. e) Folding of host rocks of the iron ore and intrusion. f) Deformation of the biotite schist and migmatite with undeformed granitoid. g) Baked contact between granitic intrusion and biotite schist. h) Photomicrograph of granitoid showing the major minerals of quartz, plagioclase, K-feldspar and biotite.


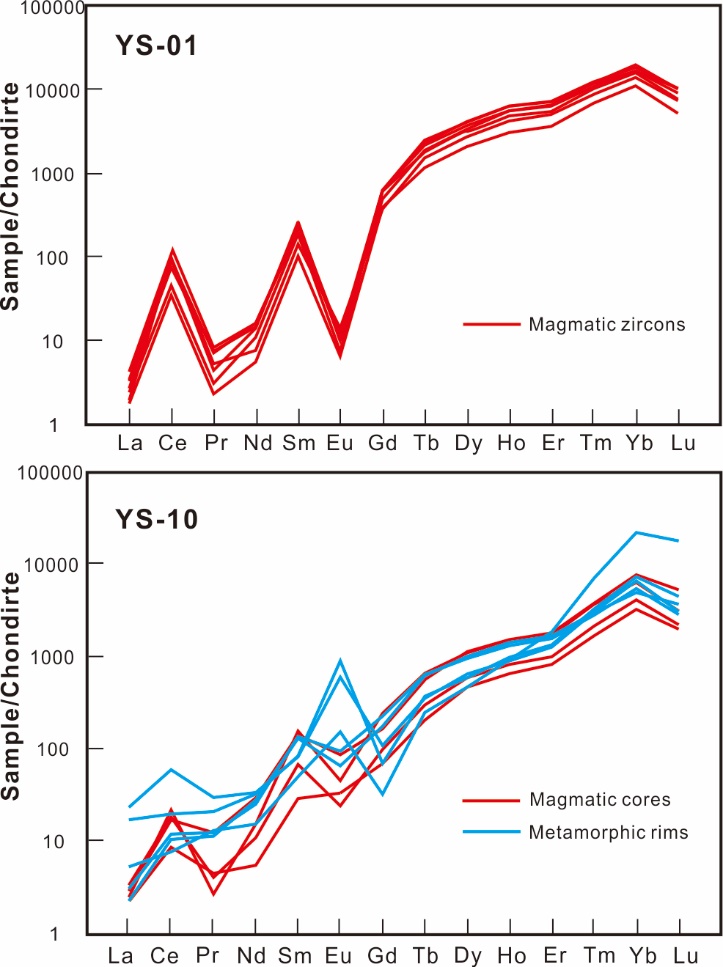


**Figure S2**. The rare earth elements (REEs) patterns of zircons for leucosome (YS-10) and granitoid (YS-01) from The Yingshan deposit, respectively.

## Tables of Supplementary Material

**Table S1**. Fe isotope composition and major element content for samples from the Yingshan deposit.

| **Yingshan iron deposit** | TFe_2_O_3_ | SiO_2_ | Al_2_O_3_ | TiO_2_ | SiO_2_+TFe_2_O_3_ | Fe/Ti | δ^56^Fe | 2SD/SE | δ^57^Fe | 2SD/SE | N |
| --- | --- | --- | --- | --- | --- | --- | --- | --- | --- | --- | --- |
|  | (wt%) | （wt%) | （wt%) | （wt%) | (wt%) | (atom) | (‰) | (‰) | (‰) | (‰) |  |
| YS-01 granitoid | 3.61 | 67.9 | 15.9 | 0.48 | 71.5 | 7.52 | 0.18 | 0.03 | 0.19 | 0.17 | 3 |
| YS-06 amphibolite | 15.8 | 50.9 | 13.5 | 1.50 | 66.6 | 10.5 | 0.08 | 0.11 | 0.18 | 0.11 | 3 |
| YS-08 leucosome | 2.04 | 77.7 | 11.5 | 0.07 | 79.7 | 29.1 | 0.47 | 0.03 | 0.71 | 0.20 | 3 |
| YS-9-1 leucosome | 1.32 | 75.6 | 13.7 | 0.14 | 77.0 | 9.43 | 0.26 | 0.11 | 0.38 | 0.08 | 3 |
| YS-9-2 leucosome | 2.35 | 75.5 | 13.2 | 0.25 | 77.8 | 9.40 | 0.13 | 0.01 | 0.22 | 0.22 | 2 |
| YS-10 leucosome | 0.97 | 75.0 | 14.1 | 0.08 | 75.9 | 12.1 | 0.14 | 0.06 | 0.16 | 0.29 | 2 |
| YS-30 granitoid | 4.01 | 69.5 | 15.1 | 0.40 | 73.5 | 10.0 | 0.00 | 0.02 | 0.02 | 0.03 | 2 |
| YS-13 ores | 50.1 | 43.7 | 3.29 | 0.11 | 93.8 | 455 | -0.16 | 0.04 | -0.20 | 0.09 | 2 |
| YS-14 ores | 54.1 | 41.3 | 2.00 | 0.05 | 95.4 | 1083 | -0.21 | 0.00 | -0.29 | 0.09 | 2 |
| YS-18 ores | 30.1 | 52.5 | 9.81 | 0.21 | 82.6 | 143 | 0.00 | 0.06 | 0.03 | 0.11 | 2 |
| YS-20 ores | 30.1 | 56.4 | 7.64 | 0.19 | 86.5 | 158 | -0.06 | 0.02 | -0.11 | 0.15 | 2 |
| YS-31 ores | 58.7 | 33.1 | 2.74 | 0.68 | 91.8 | 86.4 | -0.19 | 0.10 | -0.25 | 0.22 | 2 |
| YS-32 ores | 53.5 | 41.2 | 1.9 | 0.07 | 94.7 | 764 | -0.14 | 0.02 | -0.18 | 0.10 | 2 |
| YS-33 ores | 49.7 | 45 | 1.66 | 0.05 | 94.7 | 994 | 0.03 | 0.01 | 0.09 | 0.19 | 2 |
| YS-34 ores | 17.7 | 73.9 | 3.04 | 0.11 | 91.6 | 161 | 0.00 | 0.00 | 0.06 | 0.15 | 2 |
| YS-35 ores | 33.6 | 54.9 | 6.34 | 0.1 | 88.5 | 336 | -0.36 | 0.11 | -0.53 | 0.05 | 2 |
| YS-36 ores | 52.1 | 42.3 | 2.63 | 0.08 | 94.4 | 651 | -0.22 | 0.05 | -0.28 | 0.05 | 2 |
| YS-37 ores | 41.9 | 47.5 | 5.24 | 0.13 | 89.4 | 322 | 0.32 | 0.07 | 0.54 | 0.18 | 2 |
| YS-42 ores | 55.6 | 40.1 | 2.06 | 0.08 | 95.7 | 695 | -0.24 | 0.04 | -0.29 | 0.07 | 2 |
| YS-43 ores | 51.0 | 44.3 | 2.01 | 0.04 | 95.3 | 1275 | -0.33 | 0.08 | -0.46 | 0.06 | 2 |
| YS-50 ores | 31.0 | 48.4 | 6.81 | 0.86 | 79.4 | 36.1 | -0.04 | 0.06 | -0.02 | 0.03 | 2 |
| YS-51 ores | 48.8 | 46.4 | 1.84 | 0.08 | 95.2 | 610 | -0.07 | 0.11 | -0.06 | 0.14 | 2 |
| YS-52 ores | 34.9 | 45.8 | 5.84 | 0.65 | 80.7 | 53.7 | 0.04 | 0.03 | 0.10 | 0.04 | 1 |

**Note**: “BSE” in fig. 3 is represent the Basic Silicate Earth. The value of BSE was from^14^ and ^15^, respectively.

**Table S2**. Compilation of Fe isotope composition and major element content for igneous samples from literatures.

| **Igenous samples** | δ^56^Fe | 2SD/SE | TFe_2_O_3_ | TiO_2_ | Fe/Ti | References | |
| --- | --- | --- | --- | --- | --- | --- | --- |
|  | (‰) |  | wt.% | wt.% | (atom) | Major element | iron isotope |
| J-5 Porphyry (rhyolite) dike | 0.25 | 0.06 | 0.71 | 0.07 | 10.1 | 16 | 17 |
| J-99 Granite | 0.14 | 0.08 | 0.88 | 0.12 | 7.33 |  |  |
| J-101 Granodiorite | 0.00 | 0.02 | 3.42 | 0.54 | 6.33 | “ ” | “ ” |
| J-9 Porphyry (rhyolite) dike | 0.09 | 0.01 | 1.61 | 0.21 | 7.67 | “ ” | “ ” |
| QC-56 Granite | 0.15 | 0.07 | 0.89 | 0.11 | 8.09 | “ ” | “ ” |
| QC-8 Granite | 0.19 | 0.04 | 0.68 | 0.10 | 6.80 | “ ” | “ ” |
| J-37 Granite | 0.30 | 0.01 | 0.79 | 0.08 | 9.88 | “ ” | “ ” |
| J-13 Granite | 0.10 | 0.07 | 1.98 | 0.36 | 5.50 | “ ” | “ ” |
| QC-32C Peralkaline granite | 0.27 | 0.05 | 0.84 | 0.15 | 5.60 | “ ” | “ ” |
| QC-44 Granodiorite | 0.09 | 0.03 | 4.59 | 0.66 | 6.95 | “ ” | “ ” |
| SEG 03 01 Rhyolite | 0.08 | 0.04 | 3.40 | 0.53 | 6.42 | 18 | “ ” |
| SEG 03 03 Dacitic lava flow | 0.04 | 0.04 | 4.47 | 0.76 | 5.88 |  | “ ” |
| SEG 03 43 Rhyodacite | 0.07 | 0.02 | 4.81 | 0.83 | 5.80 | “ ” | “ ” |
| SEG 03 44 Dacitic ash flow tuff | 0.01 | 0.01 | 6.15 | 0.75 | 8.20 | “ ” | “ ” |
| SEG 03 45 Andesitic lava flow | -0.07 | 0.10 | 7.41 | 0.90 | 8.23 | “ ” | “ ” |
| SEG 03 08 Basalt | 0.00 | 0.05 | 9.83 | 0.67 | 14.7 | “ ” | “ ” |
| SB 87 56 Rhyolitic lava flow | -0.04 | 0.04 | 4.91 | 0.60 | 8.18 | “ ” | “ ” |
| SEG 03 31 Rhyolite | 0.07 | 0.02 | 3.87 | 0.48 | 8.06 | “ ” | “ ” |
| PU 03 27 Dacite | 0.05 | 0.04 | 6.95 | 0.60 | 11.6 | 19 | “ ” |
| PU 02 29 Rhyodacite | 0.08 | 0.02 | 4.98 | 0.74 | 6.73 | “ ” | “ ” |
| Puyehue-Cordon Caulle lavas 4/7 | 0.23 | 0.04 | 1.01 | 0.06 | 16.8 | 20 | “ ” |
| Puyehue-Cordon Caulle lavasCoso 5/4 | 0.31 | 0.02 | 0.89 | 0.05 | 17.8 | “ ” | “ ” |
| Puyehue-Cordon Caulle lavas 14/3 | 0.29 | 0.05 | 1.04 | 0.07 | 14.9 | “ ” | “ ” |
| Puyehue-Cordon Caulle lavas 20/5 | 0.27 | 0.05 | 1.02 | 0.09 | 11.3 | “ ” | “ ” |
| Puyehue-Cordon Caulle lavas 25/6 | 0.21 | 0.07 | 1.10 | 0.09 | 12.2 | “ ” | “ ” |
| Puyehue-Cordon Caulle lavas 26/7 | 0.04 | 0.06 | 0.88 | 0.05 | 17.6 | “ ” | “ ” |
| K-22 Rhyolite lava, Novarupta dome | 0.10 | 0.01 | 1.64 | 0.23 | 3.57 | 21 | “ ” |
| K-45 Dacite pumice, layer C | -0.02 | 0.07 | 5.80 | 0.71 | 4.08 | “ ” | “ ” |
| Bona 1 Granodiorite | 0.01 | 0.05 | 3.05 | 0.37 | 8.24 | 22 | 23 |
| Bona 2 Aplite | 0.05 | 0.05 | 1.04 | 0.11 | 9.45 |  |  |
| Siss7 Pegmatite | 0.14 | 0.05 | 0.55 | 0.05 | 11.0 | “ ” | “ ” |
| Iorio2 Tonalite | -0.04 | 0.05 | 7.62 | 0.74 | 10.3 | “ ” | “ ” |
| Sor1 Tonalite | -0.03 | 0.08 | 7.88 | 0.77 | 10.2 | “ ” | “ ” |
| Mer1 Tonalite | -0.03 | 0.05 | 7.54 | 0.75 | 10.1 | “ ” | “ ” |
| Granitoids I1 | 0.12 | 0.02 | 6.18 | 0.99 | 6.25 | 24 | 25 |
| Granitoids I2 | 0.08 | 0.02 | 4.90 | 0.58 | 8.46 | “ ” | “ ” |
| Granitoids I3 | 0.11 | 0.02 | 4.94 | 0.53 | 9.31 | “ ” | “ ” |
| Granitoids I4 | 0.19 | 0.02 | 1.71 | 0.06 | 28.4 | 25 | “ ” |
| Granitoids I5 | 0.07 | 0.02 | 5.64 | 0.54 | 10.4 | 24 | “ ” |
| Granitoids I6 | 0.06 | 0.02 | 15.1 | 0.84 | 18.0 | 25 | “ ” |
| Granitoids I7 | 0.12 | 0.02 | 3.89 | 0.42 | 9.25 | “ ” | “ ” |
| Granitoids I8 | 0.13 | 0.02 | 5.06 | 0.88 | 5.75 | 26 | “ ” |
| Granitoids I9 | 0.11 | 0.02 | 5.58 | 0.59 | 9.45 | “ ” | “ ” |
| Granitoids S1 | 0.10 | 0.02 | 4.46 | 0.54 | 8.25 | 27 | “ ” |
| Granitoids S2 | 0.10 | 0.02 | 5.28 | 0.68 | 7.76 | 26 | “ ” |
| Granitoids S3 | 0.11 | 0.02 | 5.16 | 0.64 | 8.06 | 27 | “ ” |
| Granitoids S4 | 0.16 | 0.03 | 4.54 | 0.57 | 7.97 | 28 | “ ” |
| Granitoids S5 | 0.11 | 0.02 | 4.37 | 0.53 | 8.25 | 27 | “ ” |
| Granitoids S6 | 0.14 | 0.02 | 2.07 | 0.22 | 9.42 | “ ” | “ ” |
| Granitoids S7 | 0.11 | 0.02 | 2.69 | 0.17 | 15.8 | 25 | “ ” |
| Granitoids S8 | 0.15 | 0.02 | 2.68 | 0.36 | 7.45 | 26 | “ ” |
| Granitoids S9 | 0.07 | 0.02 | 4.83 | 0.61 | 7.91 | 24 | “ ” |
| Granitoids A1 | 0.15 | 0.02 | 2.90 | 0.38 | 7.63 | 29 | “ ” |
| Granitoids A2 | 0.31 | 0.02 | 1.57 | 0.15 | 10.4 | “ ” | “ ” |
| Granitoids HP49-A | 0.16 | 0.05 | 1.46 | 0.02 | 72.8 | 30 | “ ” |
| Granitoids HP21-C | 0.24 | 0.04 | 1.69 | 0.16 | 10.6 | “ ” | “ ” |
| Pegmattites 81 BH 5-1 | 0.27 | 0.05 | 1.52 | 0.06 | 25.3 | 31 | “ ” |
| Pegmattites 81 BH 6-3 | 0.15 | 0.05 | 1.03 | 0.01 | 103 | “ ” | “ ” |
| Pegmattites WC-9 | 0.21 | 0.05 | 0.41 | 0.50 | 0.82 | “ ” | “ ” |
| Pegmattites 81 BH 9-2 | 0.20 | 0.05 | 2.44 | 0.02 | 122 | 32 | “ ” |
| Pegmattites 81 BH 10-3 | 0.22 | 0.05 | 1.71 | 0.02 | 85.6 | “ ” | “ ” |
| Pegmattites 81 BH 43-1 | 0.39 | 0.05 | 0.57 | 0.08 | 7.08 | “ ” | “ ” |
| Pegmattites P389734 | 0.19 | 0.04 | 0.56 | 0.45 | 1.24 | 33 | “ ” |
| Pegmattites P389910 | 0.06 | 0.04 | 0.57 | 0.64 | 0.89 | “ ” | “ ” |
| Pegmattites P389913 | 0.14 | 0.03 | 0.46 | 0.74 | 0.62 | “ ” | “ ” |
| Pegmattites P389726 | -0.07 | 0.03 | 0.27 | 0.91 | 0.30 | “ ” | “ ” |
| Migmatite 115-1L | 0.25 | 0.03 | 0.69 | 0.41 | 1.69 | 34 | “ ” |
| Migmatite 115-1M | 0.14 | 0.03 | 7.77 | 0.14 | 55.5 | “ ” | “ ” |
| Migmatite 118-1L | 0.27 | 0.03 | 0.50 | 3.84 | 0.13 | “ ” | “ ” |
| Migmatite 118-1M | 0.08 | 0.03 | 12.7 | 0.27 | 47.0 | “ ” | “ ” |
| Migmatite 131-1B-L | 0.28 | 0.03 | 0.06 | 0.09 | 0.68 | “ ” | “ ” |
| Migmatite 131-1B-M | 0.24 | 0.03 | 8.39 | 0.06 | 140 | “ ” | “ ” |
| Migmatite 127-1L | 0.36 | 0.03 | 0.40 | 0.16 | 2.51 | “ ” | “ ” |
| Migmatite 127-1M | 0.30 | 0.03 | 17.01 | 0.09 | 189 | “ ” | “ ” |
| Migmatite 129-1B-L | 0.28 | 0.03 | 0.55 | 0.04 | 13.8 | “ ” | “ ” |
| Migmatite 129-1B-M | 0.19 | 0.03 | 9.74 | 0.10 | 97.4 | “ ” | “ ” |
| Migmatite 118-2 | 0.48 | 0.03 | 0.82 | 0.37 | 2.22 | “ ” | “ ” |
| Migmatite 84-1 | 0.15 | 0.03 | 5.21 | 0.16 | 32.5 | “ ” | “ ” |
| Migmatite 157-1 | 0.16 | 0.03 | 6.23 | 0.17 | 36.6 | “ ” | “ ” |
| granitoids SN9402 | 0.10 | 0.06 | 3.77 | 0.59 | 6.39 | “ ” | “ ” |
| granitoids PO9207 | 0.29 | 0.13 | 0.90 | 0.09 | 10.0 | “ ” | “ ” |
| granitoids 9108 | 0.18 | 0.10 | 1.15 | 0.08 | 14.4 | “ ” | “ ” |

**Table S3**. Fe isotope composition and major element content for ~2.5Ga BIFs from literatures.

| **∽2.5Ga BIF sample** | δ^56^Fe | TFe_2_O_3_ | TiO_2_ | Fe/Ti | References | |
| --- | --- | --- | --- | --- | --- | --- |
|  | (‰) | wt.% | wt.% | (atom) | Major element | iron isotope |
| **siderate-rich BIF (Transvaal)** | -2.5‰-1.2‰ |  |  |  | 35 | 36 |
| BS-1 | " " | 30.5 | <0.04 | >762 |  |  |
| BS-2 | " " | 30.1 | <0.04 | >753 | " " | " " |
| BS-3 | " " | 24.6 | <0.04 | >614 | " " | " " |
| BS-4 | " " | 38.2 | <0.04 | >955 | " " | " " |
| BS-5 | " " | 27.6 | <0.04 | >691 | " " | " " |
| BS-6 | " " | 19.1 | <0.04 | >477 | " " | " " |
| BS-7 | " " | 27.7 | <0.04 | >693 | " " | " " |
| BS-8 | " " | 30.2 | <0.04 | >755 | " " | " " |
| BS-9 | " " | 25.2 | <0.04 | >631 | " " | " " |
| BS-10 | " " | 21.4 | <0.04 | >534 | " " | " " |
| BS-11 | " " | 23.7 | <0.04 | >591 | " " | " " |
| BS-12 | " " | 43.3 | 0.04 | 1082 | " " | " " |
| BS-13 | " " | 29.8 | <0.04 | >746 | " " | " " |
| BS-14 | " " | 23.6 | <0.04 | >590 | " " | " " |
| BS-15 | " " | 29.3 | <0.04 | >732 | " " | " " |
| BS-16 | " " | 17.5 | <0.04 | >438 | " " | " " |
| BS-17 | " " | 29.1 | 0.04 | 726 | " " | " " |
| oxidation BIF (Transvaal) | " " |  |  |  |  |  |
| BMi-1 | " " | 37.0 | <0.04 | >925 | " " | " " |
| BMi-2 | " " | 54.0 | <0.04 | >1351 | " " | " " |
| BMi-3 | " " | 48.0 | <0.04 | >1199 | " " | " " |
| BH-4 | " " | 34.8 | <0.04 | >868 | " " | " " |
| BH-5 | " " | 34.0 | <0.04 | >851 | " " | " " |
| BH-6 | " " | 46.4 | <0.04 | >1161 | " " | " " |
| BM-7 | " " | 38.8 | <0.04 | >968 | " " | " " |
| BM-8 | " " | 36.8 | <0.04 | >921 | " " | " " |
| BM-9 | " " | 47.9 | <0.04 | >1196 | " " | " " |
| BM-10 | " " | 44.6 | <0.04 | >1116 | " " | " " |
| **shaly BIF (Hamersley)** | -2.2‰-1.53‰ |  |  |  | 37 | 38,39 |
| DDH-47A* 145.8 | " " | 70.9 | 0.03 | 2363 |  |  |
| DDH-47A* 147.9 | " " | 39.1 | 0.02 | 1955 | " " | " " |
| DDH-47A* 160.4 | " " | 43.4 | 0.01 | 4340 | " " | " " |
| DDH-47A* 163.4 | " " | 78.8 | 0.01 | 7880 | " " | " " |
| Altered BIF (Hamersley) | " " |  |  |  | " " | " " |
| DDH-73 228.2 | " " | 38.8 | 0.01 | 3880 | " " | " " |
| DDH-73 232.0 | " " | 61.3 | 0.01 | 6130 | " " | " " |
| DDH-73 233.6 | " " | 58.6 | 0.01 | 5860 | " " | " " |
| DDH-86 | " " | 73.5 | 0.01 | 7350 | " " | " " |
| DDH-86 | " " | 51.1 | 0.01 | 5110 | " " | " " |
| DDH-86 | " " | 58.0 | 0.01 | 5800 | " " | " " |
| DDH-86 | " " | 53.7 | 0.01 | 5370 | " " | " " |

**Table S4**. Rare earth element (REEs) composition for ore samples from the Yingshan deposit of this study compared with those of ~2.5Ga BIF samples from literatures.

| **Sample** | **YS14** | **YS32** | **YS42** | **YS43** | **average** | **Hamerslry BIF ^(1)^** | **Kuruman BIF ^(1)^** | **Penge BIF^(1)^** |
| --- | --- | --- | --- | --- | --- | --- | --- | --- |
| La | 8.97 | 6.26 | 9.40 | 8.93 | 8.39 | 2.22 | 1.80 | 6.86 |
| Ce | 17.3 | 11.6 | 17.6 | 17.1 | 15.9 | 3.43 | 3.04 | 12.7 |
| Pr | 2.16 | 1.44 | 2.29 | 2.23 | 2.03 | - | 0.35 | 1.47 |
| Nd | 8.35 | 5.71 | 9.02 | 9.10 | 8.05 | 1.77 | 1.62 | 5.66 |
| Sm | 1.59 | 1.18 | 1.85 | 2.02 | 1.66 | 0.35 | 0.36 | 1.13 |
| Eu | 0.49 | 0.38 | 0.55 | 0.64 | 0.52 | 0.15 | 0.15 | 0.34 |
| Gd | 1.65 | 1.34 | 1.93 | 2.13 | 1.76 | 0.51 | 0.47 | 1.15 |
| Tb | 0.29 | 0.27 | 0.36 | 0.39 | 0.33 | - | 0.08 | 0.19 |
| Dy | 1.67 | 1.78 | 2.16 | 2.37 | 2.00 | 0.52 | 0.55 | 1.17 |
| Y | 12.2 | 14.1 | 15.4 | 16.8 | 14.6 | - | 6.35 | 10.2 |
| Ho | 0.36 | 0.42 | 0.45 | 0.51 | 0.44 | - | 0.14 | 0.26 |
| Er | 0.91 | 1.10 | 1.15 | 1.28 | 1.11 | 0.41 | 0.46 | 0.85 |
| Tm | 0.13 | 0.17 | 0.16 | 0.19 | 0.16 | - | 0.07 | 0.12 |
| Yb | 0.79 | 1.03 | 0.99 | 1.11 | 0.98 | 0.41 | 0.49 | 0.85 |
| Lu | 0.14 | 0.17 | 0.16 | 0.19 | 0.16 | 0.07 | 0.09 | 0.13 |
| ΣREE+Y | 57.0 | 47.0 | 63.4 | 65.0 | 58.1 | 9.84 | 16.0 | 43.1 |
| HREE/LREE | 1.86 | 2.91 | 2.12 | 2.24 | 2.28 | 1.74 | 3.72 | 2.17 |
| Y/Y* | 1.26 | 1.30 | 1.23 | 1.23 | 1.26 | - | 1.82 | 1.85 |
| Y/Ho^(2)^ | 34.0 | 33.7 | 33.8 | 33.1 | 33.7 | - | 45.1 | 46.1 |
| La/La* | 1.22 | 1.27 | 1.31 | 1.29 | 1.27 | 1.77 | 1.31 | 1.42 |
| Eu/Eu* | 1.42 | 1.41 | 1.38 | 1.46 | 1.42 | 1.61 | 1.70 | 1.63 |
| Ce/Ce* | 0.91 | 0.89 | 0.87 | 0.88 | 0.89 | - | 0.88 | 0.85 |

**Notes**: (Y/Y*) _SN_ = [2Y/(Dy + Ho)]_SN_; (Ce/Ce*)_SN_ = [Ce/(La*Pr)^0.5^]_SN_; (La/La*) _SN_ = [La/(la/(Ce*Ce/Pr))]_SN_; (Eu/Eu*) _SN_ = [Eu/(Sm*Tb)^0.5^]_SN_;

(1) The REEs+Y compistion of ~2.5Ga BIF (Hamersely, Kuruman and Peneg) is the avergae valve form the previous study on Hamersely basin^40^ and Transvval basin^41^.

(2)Y/Ho value showed with the atomic ratio;

(3)The Pro-Archean Austrian Shale (PAAS) data was from^42^.

**Table S5**. U-Pb isotope composition of zircons for leucosome (YS-10) and granitoid (YS-01) of this study.

| Spot | Isotopic ratios | | | | | | | Age(Ma) | | | | | |
| --- | --- | --- | --- | --- | --- | --- | --- | --- | --- | --- | --- | --- | --- |
|  | ^207^Pb/^206^Pb | 1σ | ^207^Pb/^235^U | 1σ | ^206^Pb/^238^U | 1σ | Th/U | ^207^Pb/^206^Pb | 1σ | ^207^Pb/^235^U | 1σ | ^206^Pb/^238^U | 1σ |
| Leucosome (YS10) | |  |  |  |  |  |  |  |  |  |  |  |  |
| 01 | 0.0759 | 0.0023 | 0.550 | 0.015 | 0.0526 | 0.0006 | 0.03 | 1093 | 61 | 445 | 10 | 330 | 4 |
| 02 | 0.1474 | 0.0043 | 6.321 | 0.168 | 0.3110 | 0.0039 | 0.39 | 2316 | 51 | 2021 | 23 | 1746 | 19 |
| 03 | 0.1167 | 0.0031 | 1.407 | 0.034 | 0.0875 | 0.0010 | 0.05 | 1906 | 48 | 892 | 14 | 540 | 6 |
| 04 | 0.1131 | 0.0033 | 1.349 | 0.036 | 0.0865 | 0.0010 | 0.06 | 1850 | 54 | 867 | 16 | 535 | 6 |
| 05 | 0.1603 | 0.0033 | 10.268 | 0.212 | 0.4647 | 0.0058 | 0.50 | 2459 | 36 | 2459 | 19 | 2460 | 25 |
| 06 | 0.1447 | 0.0033 | 3.561 | 0.072 | 0.1785 | 0.0020 | 0.15 | 2284 | 41 | 1541 | 16 | 1059 | 11 |
| 07 | 0.1607 | 0.0047 | 10.257 | 0.296 | 0.4638 | 0.0068 | 0.75 | 2463 | 50 | 2458 | 27 | 2456 | 30 |
| 08 | 0.1471 | 0.0039 | 3.587 | 0.087 | 0.1769 | 0.0021 | 0.22 | 2312 | 47 | 1547 | 19 | 1050 | 11 |
| 09 | 0.1400 | 0.0026 | 3.926 | 0.071 | 0.2037 | 0.0023 | 0.42 | 2227 | 33 | 1619 | 15 | 1195 | 12 |
| 10 | 0.1444 | 0.0056 | 2.930 | 0.106 | 0.1472 | 0.0021 | 0.16 | 2280 | 69 | 1390 | 27 | 885 | 12 |
| 11 | 0.1600 | 0.0035 | 10.212 | 0.224 | 0.4639 | 0.0059 | 0.51 | 2455 | 38 | 2454 | 20 | 2457 | 26 |
| 12 | 0.1489 | 0.0041 | 4.217 | 0.105 | 0.2054 | 0.0024 | 0.22 | 2333 | 48 | 1677 | 20 | 1204 | 13 |
| 13 | 0.1565 | 0.0032 | 8.628 | 0.173 | 0.4003 | 0.0047 | 0.68 | 2419 | 35 | 2299 | 18 | 2170 | 22 |
| 14 | 0.1553 | 0.0049 | 5.964 | 0.173 | 0.2786 | 0.0036 | 0.43 | 2405 | 55 | 1971 | 25 | 1584 | 18 |
| 15 | 0.1636 | 0.0032 | 8.925 | 0.173 | 0.3961 | 0.0048 | 0.47 | 2493 | 34 | 2330 | 18 | 2151 | 22 |
| 16 | 0.1659 | 0.0033 | 9.941 | 0.196 | 0.4350 | 0.0051 | 0.37 | 2516 | 35 | 2429 | 18 | 2328 | 23 |
| 17 | 0.0731 | 0.0021 | 0.475 | 0.012 | 0.0472 | 0.0005 | 0.06 | 1016 | 59 | 395 | 8 | 297 | 3 |
| 18 | 0.1601 | 0.0041 | 10.262 | 0.254 | 0.4663 | 0.0057 | 0.43 | 2457 | 44 | 2459 | 23 | 2467 | 25 |
| 19 | 0.1314 | 0.0034 | 3.299 | 0.075 | 0.1821 | 0.0022 | 0.04 | 2117 | 46 | 1481 | 18 | 1078 | 12 |
| 20 | 0.1633 | 0.0047 | 9.628 | 0.275 | 0.4291 | 0.0062 | 0.45 | 2490 | 50 | 2400 | 26 | 2302 | 28 |
| 21 | 0.1656 | 0.0033 | 8.838 | 0.174 | 0.3875 | 0.0047 | 0.68 | 2514 | 34 | 2321 | 18 | 2111 | 22 |
| 22 | 0.0892 | 0.0022 | 0.915 | 0.020 | 0.0744 | 0.0008 | 0.04 | 1408 | 48 | 660 | 10 | 463 | 5 |
| 23 | 0.1551 | 0.0036 | 5.261 | 0.121 | 0.2463 | 0.0033 | 0.66 | 2403 | 41 | 1862 | 20 | 1419 | 17 |
| 24 | 0.0801 | 0.0038 | 0.529 | 0.024 | 0.0479 | 0.0007 | 0.03 | 1199 | 96 | 431 | 16 | 302 | 4 |
| 25 | 0.1633 | 0.0057 | 10.337 | 0.357 | 0.4605 | 0.0078 | 0.36 | 2491 | 60 | 2465 | 32 | 2442 | 34 |
| 26 | 0.1644 | 0.0034 | 9.948 | 0.206 | 0.4388 | 0.0054 | 0.46 | 2502 | 36 | 2430 | 19 | 2345 | 24 |
| 27 | 0.0761 | 0.0021 | 0.614 | 0.015 | 0.0585 | 0.0007 | 0.02 | 1099 | 55 | 486 | 9 | 366 | 4 |
| 28 | 0.1601 | 0.0040 | 10.233 | 0.248 | 0.4644 | 0.0058 | 0.42 | 2457 | 43 | 2456 | 22 | 2459 | 25 |
| 29 | 0.1412 | 0.0038 | 2.812 | 0.068 | 0.1444 | 0.0017 | 0.17 | 2242 | 48 | 1359 | 18 | 870 | 10 |
| 30 | 0.0925 | 0.0023 | 0.957 | 0.021 | 0.0751 | 0.0008 | 0.05 | 1478 | 49 | 682 | 11 | 467 | 5 |
| 31 | 0.1591 | 0.0031 | 8.149 | 0.158 | 0.3721 | 0.0045 | 0.68 | 2446 | 34 | 2248 | 18 | 2039 | 21 |
| 32 | 0.1525 | 0.0032 | 8.093 | 0.168 | 0.3854 | 0.0049 | 0.58 | 2374 | 36 | 2241 | 19 | 2101 | 23 |
| 33 | 0.1181 | 0.0030 | 1.324 | 0.029 | 0.0813 | 0.0010 | 0.05 | 1928 | 46 | 856 | 13 | 504 | 6 |
| 34 | 0.1607 | 0.0032 | 9.230 | 0.180 | 0.4167 | 0.0049 | 0.44 | 2463 | 34 | 2361 | 18 | 2245 | 22 |
| 35 | 0.0950 | 0.0024 | 0.924 | 0.021 | 0.0705 | 0.0008 | 0.04 | 1529 | 49 | 664 | 11 | 439 | 5 |
| 36 | 0.1008 | 0.0030 | 1.041 | 0.029 | 0.0749 | 0.0009 | 0.07 | 1639 | 57 | 724 | 14 | 465 | 5 |
| 37 | 0.1605 | 0.0037 | 10.202 | 0.237 | 0.4618 | 0.0062 | 0.67 | 2461 | 40 | 2453 | 21 | 2447 | 27 |
| 38 | 0.1495 | 0.0042 | 5.272 | 0.142 | 0.2565 | 0.0032 | 0.44 | 2340 | 49 | 1864 | 23 | 1472 | 16 |
| 39 | 0.1487 | 0.0057 | 4.960 | 0.177 | 0.2419 | 0.0034 | 0.38 | 2331 | 67 | 1813 | 30 | 1397 | 18 |
| 40 | 0.1484 | 0.0030 | 4.989 | 0.100 | 0.2441 | 0.0030 | 0.47 | 2327 | 35 | 1817 | 17 | 1408 | 15 |
| 41 | 0.1483 | 0.0048 | 5.692 | 0.166 | 0.2784 | 0.0038 | 0.53 | 2327 | 56 | 1930 | 25 | 1583 | 19 |
| 42 | 0.1686 | 0.0042 | 11.254 | 0.277 | 0.4845 | 0.0067 | 0.53 | 2544 | 43 | 2544 | 23 | 2547 | 29 |
| 43 | 0.0800 | 0.0021 | 0.590 | 0.014 | 0.0535 | 0.0006 | 0.04 | 1198 | 52 | 471 | 9 | 336 | 4 |
| 44 | 0.1519 | 0.0036 | 8.262 | 0.193 | 0.3948 | 0.0048 | 0.48 | 2367 | 42 | 2260 | 21 | 2145 | 22 |
| 45 | 0.1611 | 0.0033 | 9.085 | 0.179 | 0.4091 | 0.0046 | 0.58 | 2468 | 35 | 2347 | 18 | 2211 | 21 |
| 46 | 0.1498 | 0.0047 | 4.797 | 0.137 | 0.2322 | 0.0030 | 0.33 | 2344 | 55 | 1784 | 24 | 1346 | 16 |
| 47 | 0.0723 | 0.0036 | 0.399 | 0.019 | 0.0401 | 0.0006 | 0.57 | 993 | 105 | 341 | 14 | 253 | 4 |
| 48 | 0.0797 | 0.0024 | 0.443 | 0.012 | 0.0403 | 0.0005 | 0.03 | 1191 | 61 | 372 | 9 | 255 | 3 |
| 49 | 0.1620 | 0.0037 | 10.222 | 0.232 | 0.4584 | 0.0058 | 0.57 | 2477 | 40 | 2455 | 21 | 2432 | 26 |
| 50 | 0.1363 | 0.0025 | 3.381 | 0.061 | 0.1800 | 0.0020 | 0.37 | 2181 | 33 | 1500 | 14 | 1067 | 11 |
| 51 | 0.1307 | 0.0027 | 1.439 | 0.029 | 0.0800 | 0.0009 | 0.56 | 2107 | 38 | 906 | 12 | 496 | 6 |
| 52 | 0.1619 | 0.0043 | 10.252 | 0.273 | 0.4605 | 0.0069 | 0.46 | 2475 | 46 | 2458 | 25 | 2442 | 30 |
| 53 | 0.1602 | 0.0059 | 10.170 | 0.370 | 0.4642 | 0.0082 | 0.48 | 2458 | 63 | 2450 | 34 | 2458 | 36 |
| 54 | 0.0953 | 0.0039 | 0.830 | 0.031 | 0.0631 | 0.0010 | 0.03 | 1534 | 79 | 613 | 17 | 395 | 6 |
| 55 | 0.1599 | 0.0029 | 9.210 | 0.164 | 0.4181 | 0.0048 | 0.77 | 2454 | 31 | 2359 | 16 | 2252 | 22 |
| 56 | 0.0756 | 0.0034 | 0.417 | 0.018 | 0.0400 | 0.0005 | 0.01 | 1084 | 93 | 354 | 13 | 253 | 3 |
| 57 | 0.1610 | 0.0032 | 9.412 | 0.184 | 0.4243 | 0.0052 | 0.57 | 2466 | 34 | 2379 | 18 | 2280 | 24 |
| 58 | 0.0818 | 0.0041 | 0.469 | 0.023 | 0.0416 | 0.0006 | 0.23 | 1240 | 101 | 390 | 16 | 263 | 4 |
| 59 | 0.1620 | 0.0055 | 10.321 | 0.351 | 0.4626 | 0.0085 | 0.55 | 2477 | 59 | 2464 | 32 | 2451 | 37 |
| 60 | 0.0834 | 0.0022 | 0.634 | 0.015 | 0.0551 | 0.0006 | 0.04 | 1278 | 53 | 498 | 9 | 346 | 4 |
| 61 | 0.1531 | 0.0033 | 5.551 | 0.114 | 0.2632 | 0.0030 | 0.49 | 2380 | 37 | 1908 | 18 | 1506 | 15 |
| 62 | 0.1608 | 0.0050 | 10.196 | 0.313 | 0.4616 | 0.0075 | 0.37 | 2464 | 53 | 2453 | 28 | 2446 | 33 |
| 63 | 0.1526 | 0.0058 | 4.155 | 0.148 | 0.1975 | 0.0027 | 0.24 | 2375 | 66 | 1665 | 29 | 1162 | 14 |
| 64 | 0.1613 | 0.0036 | 8.681 | 0.196 | 0.3913 | 0.0053 | 0.58 | 2469 | 39 | 2305 | 21 | 2129 | 24 |
| 65 | 0.1609 | 0.0052 | 10.153 | 0.332 | 0.4584 | 0.0085 | 0.43 | 2465 | 56 | 2449 | 30 | 2433 | 38 |
| 66 | 0.1605 | 0.0035 | 10.235 | 0.221 | 0.4625 | 0.0060 | 0.73 | 2461 | 38 | 2456 | 20 | 2451 | 26 |
| 67 | 0.1387 | 0.0026 | 4.647 | 0.087 | 0.2431 | 0.0028 | 0.53 | 2211 | 34 | 1758 | 16 | 1403 | 15 |
| 68 | 0.1003 | 0.0026 | 1.213 | 0.028 | 0.0877 | 0.0010 | 0.06 | 1629 | 48 | 807 | 13 | 542 | 6 |
| Granitoid (YS01) | |  |  |  |  |  |  |  |  |  |  |  |  |
| 69 | 0.0658 | 0.0017 | 1.194 | 0.030 | 0.1316 | 0.0016 | 1.31 | 801 | 54 | 798 | 14 | 797 | 9 |
| 70 | 0.0659 | 0.0019 | 1.210 | 0.034 | 0.1334 | 0.0017 | 1.71 | 804 | 61 | 805 | 16 | 807 | 9 |
| 71 | 0.0664 | 0.0023 | 1.206 | 0.042 | 0.1320 | 0.0020 | 1.49 | 820 | 75 | 803 | 19 | 799 | 11 |
| 72 | 0.0664 | 0.0023 | 1.237 | 0.041 | 0.1352 | 0.0018 | 1.42 | 819 | 72 | 817 | 19 | 817 | 10 |
| 73 | 0.0670 | 0.0031 | 1.213 | 0.055 | 0.1315 | 0.0023 | 1.12 | 837 | 99 | 806 | 25 | 796 | 13 |
| 74 | 0.0658 | 0.0019 | 1.210 | 0.034 | 0.1335 | 0.0016 | 1.64 | 800 | 61 | 805 | 15 | 808 | 9 |
| 75 | 0.0663 | 0.0016 | 1.217 | 0.029 | 0.1335 | 0.0016 | 1.70 | 814 | 51 | 809 | 13 | 808 | 9 |
| 76 | 0.0661 | 0.0016 | 1.225 | 0.029 | 0.1345 | 0.0016 | 1.39 | 809 | 51 | 812 | 13 | 813 | 9 |
| 77 | 0.0663 | 0.0018 | 1.226 | 0.033 | 0.1342 | 0.0017 | 1.53 | 817 | 58 | 812 | 15 | 812 | 10 |
| 78 | 0.0670 | 0.0062 | 1.221 | 0.110 | 0.1327 | 0.0035 | 0.88 | 838 | 199 | 810 | 50 | 803 | 20 |
| 79 | 0.0661 | 0.0020 | 1.213 | 0.036 | 0.1334 | 0.0017 | 1.46 | 810 | 64 | 807 | 16 | 807 | 10 |
| 80 | 0.0671 | 0.0022 | 1.231 | 0.040 | 0.1332 | 0.0017 | 1.19 | 839 | 71 | 815 | 18 | 806 | 10 |
| 81 | 0.0661 | 0.0019 | 1.221 | 0.034 | 0.1341 | 0.0017 | 1.35 | 810 | 61 | 810 | 16 | 811 | 10 |
| 82 | 0.0667 | 0.0019 | 1.213 | 0.034 | 0.1321 | 0.0017 | 1.46 | 829 | 60 | 807 | 15 | 800 | 10 |
| 83 | 0.0661 | 0.0021 | 1.215 | 0.038 | 0.1337 | 0.0018 | 1.36 | 809 | 68 | 808 | 17 | 809 | 10 |
| 84 | 0.0662 | 0.0033 | 1.219 | 0.059 | 0.1339 | 0.0022 | 1.53 | 814 | 106 | 809 | 27 | 810 | 13 |
| 85 | 0.0657 | 0.0018 | 1.192 | 0.033 | 0.1318 | 0.0016 | 2.00 | 797 | 60 | 797 | 15 | 798 | 9 |
| 86 | 0.0662 | 0.0027 | 1.203 | 0.049 | 0.1321 | 0.0019 | 1.18 | 813 | 89 | 802 | 22 | 800 | 11 |

**Table S6**. Trace element composition (ppm) of zircons for leucosome (YS-10) and granitoid (YS-01) from The Yingshan deposit.

| **YS-10** | **La** | **Ce** | **Pr** | **Nd** | **Sm** | **Eu** | **Gd** | **Tb** | **Dy** | **Ho** | **Er** | **Tm** | **Yb** | **Lu** | **Th** | **U** | **Th/U** |
| --- | --- | --- | --- | --- | --- | --- | --- | --- | --- | --- | --- | --- | --- | --- | --- | --- | --- |
| **5** | 0.54 | 6.46 | 1.08 | 12.9 | 19.4 | 3.73 | 35.3 | 22.6 | 248 | 78.2 | 269 | 76.4 | 807 | 91.2 | 136 | 117 | 1.16 |
| **6** | 0.69 | 10.5 | 1.16 | 13.6 | 21.3 | 4.97 | 33.1 | 21.0 | 281 | 83.1 | 290 | 94.3 | 1246 | 131 | 161 | 358 | 0.45 |
| **23** | 0.54 | 5.32 | 0.42 | 2.53 | 4.33 | 1.89 | 13.9 | 7.45 | 116 | 36.0 | 132 | 41.8 | 527 | 49.1 | 88.7 | 91.8 | 0.97 |
| **25** | 1.26 | 4.75 | 1.22 | 7.17 | 7.46 | 8.69 | 6.47 | 9.03 | 115 | 50.7 | 299 | 176 | 3574 | 443 | 34.5 | 39.0 | 0.88 |
| **32** | 0.79 | 11.5 | 0.38 | 5.03 | 10.1 | 1.37 | 19.8 | 10.9 | 146 | 45.2 | 160 | 53.2 | 668 | 54.8 | 110 | 131 | 0.84 |
| **48** | 4.08 | 12.2 | 1.96 | 14.9 | 12.5 | 51.06 | 14.0 | 13.5 | 147 | 53.8 | 216 | 81.5 | 1183 | 111 | 39.2 | 530 | 0.07 |
| **50** | 0.59 | 13.5 | 0.25 | 7.07 | 23.3 | 2.57 | 48.8 | 24.0 | 276 | 84.1 | 269 | 91.5 | 1029 | 77.3 | 217 | 292 | 0.74 |
| **52** | 0.73 | 7.38 | 1.17 | 11.62 | 20.2 | 5.36 | 45.3 | 23.3 | 237 | 72.2 | 253 | 70.9 | 876 | 72.5 | 132 | 147 | 0.90 |
| **58** | 5.55 | 36.7 | 2.79 | 15.5 | 12.3 | 33.9 | 21.8 | 12.8 | 161 | 49.5 | 204 | 75.8 | 1070 | 76.0 | 27.9 | 728 | 0.04 |
| **YS-01** | **La** | **Ce** | **Pr** | **Nd** | **Sm** | **Eu** | **Gd** | **Tb** | **Dy** | **Ho** | **Er** | **Tm** | **Yb** | **Lu** | **Th** | **U** | **Th/U** |
| **70** | 0.76 | 45.4 | 0.68 | 6.57 | 36.1 | 0.64 | 123 | 88.3 | 984 | 340 | 1134 | 286 | 2654 | 252 | 694 | 242 | 2.87 |
| **71** | 0.62 | 56.1 | 0.40 | 6.29 | 38.3 | 0.55 | 119 | 77.1 | 902 | 299 | 1002 | 266 | 2593 | 220 | 750 | 333 | 2.25 |
| **74** | 0.79 | 57.0 | 0.65 | 7.18 | 33.3 | 0.55 | 121 | 81.7 | 1003 | 339 | 1145 | 303 | 2891 | 246 | 1415 | 453 | 3.13 |
| **76** | 0.45 | 59.2 | 0.48 | 3.42 | 20.7 | 0.79 | 73.3 | 54.2 | 662 | 226 | 798 | 228 | 2210 | 179 | 1986 | 535 | 3.71 |
| **79** | 0.99 | 69.8 | 0.75 | 7.21 | 29.3 | 0.6 | 97.4 | 67.0 | 809 | 295 | 1030 | 294 | 3138 | 244 | 1589 | 566 | 2.81 |
| **82** | 0.41 | 20.9 | 0.21 | 2.48 | 14.8 | 0.36 | 76.6 | 41.6 | 502 | 165 | 574 | 168 | 1762 | 126 | 367 | 175 | 2.10 |
| **83** | 0.56 | 27.5 | 0.28 | 4.89 | 27.2 | 0.41 | 119 | 63.6 | 771 | 260 | 861 | 249 | 2516 | 185 | 555 | 242 | 2.29 |

**Note:** The Chondrite data was from^14^.

## References

1. Alkmim, F.F., & Marshak, S. Transamazonian orogeny in the Southern Sao Francisco craton region, Minas Gerais, Brazil: evidence for Paleoproterozoic collision and collapse in the Quadrilátero Ferrıfero. *Precambrian Research* **90**, 29–58, doi: 10.1016/S0301-9268(98)00032-1 (1998).
2. Li, Y. et al. Petrogenesis and tectonic implications of Paleoproterozoic metapelitic rocks in the Archean Kongling Complex from the northern Yangtze Craton, South China. *Precambrian Research* **276**, 158-177, doi:10.1016/j.precamres.2016.01.028 (2016).
3. Yin et al. 2.1-1.85Ga tectonic events in the Yangtze Block, South China: petrological and geochronological evidence from the Kongling Complex and implications for the reconstruction of supercontinent Columbia. *Lithos* **182**, 200-2010, doi:10.1016/j.lithos.2013.10.012 (2013).
4. Spier, C. A., de Oliveira, S. M., Sial, A. N., & Rios, F. J. Geochemistry and genesis of the banded iron formations of the Cauê Formation, Quadrilátero Ferrífero, Minas Gerais, Brazil. *Precambrian Research* 152, 170-206, doi:10.1016/j.precamres.2006.10.003 (2007).
5. Wei, J. Q., Wang, J. X., Wang, X. D., Shan, M. Y., & Guo, H. M. Dating of mafic dikes from Kongling Group in Huangling Area and its implicatons. *Journal of Northwest University (Natural Science Edition)* **3**, 016. (In Chinese)
6. Hu, J. et al. A ~2.5 Ga magmatic event at the northern margin of the Yangtze craton: Evidence from U-Pb dating and Hf isotope analysis of zircons from the Douling Complex in the South Qinling orogeny. *Chinese Science Bulletin* **58**, 3564–3579, doi: 10.1007/s11434-013-5904-1 (2013). (In Chinese)
7. Farina, F., Albert, C., & Lana, C. The Neoarchean transition between medium-and high-K granitoids: Clues from the Southern São Francisco Craton (Brazil). *Precambrian Research* **266**, 375-394, doi:10.1016/j.precamres.2015.05.038 (2015).
8. Zhou, G. et al. The 2.65 Ga A-type granite in the northeastern Yangtze craton: Petrogenesis and geological implications. *Precambrian Research* **258**, 247-259, doi:10.1016/j.precamres.2015.01.003 (2015).
9. Chen, K. et al. 2.6–2.7 Ga crustal growth in Yangtze craton, South China. *Precambrian Research* **224**, 472-490, doi:10.1016/j.precamres.2012.10.017 (2013).
10. Zhang G.W., Yu Z.P., Dong Y.P., & Yao A.P. On Precambrian framework and evolution of the Qinling belt. *Acta Petrologica Sinica* **16**, 11-21 (2000). (In Chinese)
11. Qiu, Y.M., Gao, S., McNaughton, N.J., Groves, D.I., & Ling, W.L. First evidence of ∼3.2Ga continental crust in the Yangtze craton of south China and its implications for Archean crustal evolution and Phanerozoic tectonics. *Geology* **28**, 1–14, doi: 10.1130/0091-7613(2000)​028<0011:FEOGCC>​2.0.CO;2 (2000).
12. Zhang, S. B. et al. Zircon U-Pb age and Hf-O isotope evidence for Paleoproterozoic metamorphic event in South China. *Precambrian Research* **151**, 265-288, doi:10.1016/j.precamres.2006.08.009 (2006).
13. Gao, S. et al. Age and growth of the Archean Kongling terrain, South China, with emphasis on 3.3 Ga granitoid gneisses. *American Journal of science* **311**, 153-182, doi: 10.2475/02.2011.03 (2011).
14. McDonough, W.F & Sun, S.S. The composition of the earth. *Chemical geology* **120**, 223-253, doi:10.1016/0009-2541(94)00140-4 (1995).
15. Poitrasson, F., Delpech, G., & Grégoire, M. On the iron isotope heterogeneity of lithospheric mantle xenoliths: implications for mantle metasomatism, the origin of basalts and the iron isotope composition of the Earth. *Contributions to Mineralogy and Petrology* **165**, 1243-1258, doi: 10.1007/s00410-013-0856-7 (2013).
16. Johnson C.M., Czamanske G.K., & Lipman P.W. Geochemistry of intrusive rocks associated with the Latir volcanic field, New Mexico, and contrasts between evolution of plutonic and volcanic rocks. *Contr. Mineral. Petrol.* **103**, 90-109, doi: 10.1007/BF00371367 (1989).
17. Heimann, A., Beard, B. L., & Johnson, C. M. The role of volatile exsolution and sub-solidus fluid/rock interactions in producing high 56 Fe/54 Fe ratios in siliceous igneous rocks: *Geochimica et Cosmochimica Acta* **72**, 4379-4396, doi: 10.1016/j.gca.2008.06.009 (2008).
18. Jicha B.R. et al. Rapid magma ascent and generation of 230Th excesses in the lower crust at Puyehue-Cordón Caulle, Southern Volcanic Zone, Chile. *Earth Planet. Sci, Lett* **255**, 229-242, doi:10.1016/j.epsl.2006.12.017 (2007).
19. Jicha B.R., Singer B.S., Beard B.L., & Johnson C.M. Contrasting timescales of crystallization and magma storage beneath the Aleutian Island arc. *Earth Planet. Sci. Lett.* **236**, 195-210, doi: 10.1016/j.epsl.2005.05.002 (2005).
20. Bacon C.R., Macdonald R., Smith R.L., & Baedecker P.A. Pleistocene high-silica rhyolites of the Coso volcanic field, Inyo County, California. *J. Geophys. Res* **86**, 10223-10241, doi: 10.1029/JB086iB11p10223 (1981).
21. Hildreth, W. The compositionally zoned eruption of 1912 in the valley of ten thousand smokes, Katmai National Park, Alaska. *Journal of Volcanology and Geothermal Research* **18**, 1-56, doi: 10.1016/0377-0273(83)90003-3 (1983).
22. von Blanckenburg F., Fruhgreen G., Diethelm K., & Stille P. Nd-isotopic, Sr-isotopic, O-isotopic and chemical evidence for a 2-stage contamination history of the mantle magma in the Central-Alpine Bergell intrusion. *Contr. Mineral. Petrol* **110**, 33-45, doi: 10.1007/BF00310880 (1992).
23. Schoenberg R. & von Blanckenburg F. Modes of planetary-scale Fe isotope fractionation. *Earth Planet. Sci. Lett* **252**, 342-359, doi:10.1016/j.epsl.2006.09.045 (2006).
24. Sha, L. K., & Chappell, B. W. Apatite chemical composition, determined by electron microprobe and laser-ablation inductively coupled plasma mass spectrometry, as a probe into granite petrogenesis. *Geochimica et Cosmochimica Acta* **63**, 3861-3881, doi:10.1016/S0016-7037(99)00210-0 (1999).
25. Telus, M. et al. Iron, Zinc, Magnesium and Uranium Isotopic Fractionation During Continental Crust Diferentiation: The tale from migmatites, granitoids, and pegmatites. *Geochimica et Cosmochimica Acta* **97**, 247-265 (2012).
26. Chappell, B. W., White, A. J. R., & Wyborn, D. The Cowra Granodiorite and its enclaves. Excursion guide, IAVCEI Canberra, Canberra (1993).
27. Chappell, B. W., & Simpson, P. R. Source rocks of I-and S-type granites in the lachlan fold belt, Southeastern Australia [and discussion]. *Philosophical Transactions of the Royal Society of London A. Mathematical, Physical and Engineering Sciences*, **310**, 693-707 (1984).
28. Hine, R., Williams, I. S., Chappell, B. W., & White, A. J. R. Contrasts between I‐and S‐type granitoids of the Kosciusko Batholith. *Journal of the Geological Society of Australia* **25**, 219-234, doi: 10.1080/00167617808729029 (1978).
29. King, P. L., White, A. J. R., Chappell, B. W., & Allen, C. M. Characterization and origin of aluminous A-type granites from the Lachlan Fold Belt, southeastern Australia. *Journal of petrology* **38**, 371-391, doi:10.1093/petroj/38.3.371 (1997).
30. Nabelek, P. I., Russ-Nabelek, C., & Denison, J. R. The generation and crystallization conditions of the Proterozoic Harney Peak leucogranite, Black Hills, South Dakota, USA: petrologic and geochemical constraints. *Contributions to Mineralogy and Petrology* **110**, 173-191, doi: 10.1007/BF00310737 (1992).
31. Walker R.J. Origin of the Tin Mountain Pegmatites, Black Hills, South Dakota. Ph. D. thesis, State University of New York at Stony Brook (1984).
32. Walker, R. J., Hanson, G. N., Papike, J. J., O'neil, J. R., & Laul, J. C. Internal evolution of the Tin Mountain pegmatite, Black Hills, South Dakota. *Am. Mineral* **71**, 440-459 (1986).
33. Groat, L. A. et al. Geology and mineralogy of the Little Nahanni rare-element granitic pegmatites, Northwest Territories. *The Canadian Mineralogist* **41**, 139-160, doi: 10.2113/gscanmin.41.1.139 (2003).
34. Nabelek, P. I. Trace element distribution among rock-forming minerals in Black Hills migmatites, South Dakota: A case for solid-state equilibrium. *American Mineralogist* **84**, 1256-1269, doi:10.2138/am-1999-0904 (1999).
35. Klein, C., & Beukes, N. J. Geochemistry and sedimentology of a facies transition from limestone to iron-formation deposition in the early Proterozoic Transvaal Supergroup, South Africa. *Economic Geology* **84**, 1733-1774, doi: 10.2113/gsecongeo.84.7.1733 (1989).
36. Johnson, C. M., Beard, B. L., Beukes, N. J., Klein, C., & O'Leary, J. M. Ancient geochemical cycling in the Earth as inferred from Fe isotope studies of banded iron formations from the Transvaal Craton. *Contributions to Mineralogy and Petrology* **144**, 523-547, doi: 10.1007/s00410-002-0418-x (2003).
37. Webb, A. D., Dickens, G. R., & Oliver, N. H. From banded iron-formation to iron ore: geochemical and mineralogical constraints from across the Hamersley Province, Western Australia. *Chemical Geology* **197**, p.215-251, doi:10.1016/S0009-2541(02)00352-2 (2003).
38. Steinhoefel, G., von Blanckenburg, F., Horn, I., Konhauser, K. O., Beukes, N. J., & Gutzmer, J. Deciphering formation processes of banded iron formations from the Transvaal and the Hamersley successions by combined Si and Fe isotope analysis using UV femtosecond laser ablation. *Geochimica et Cosmochimica Acta* **74**, p.2677-2696,doi: 10.1016/j.gca.2010.01.028 (2010).
39. Li, W. et al. Contrasting behavior of oxygen and iron isotopes in banded iron formations revealed by in situ isotopic analysis. *Earth and Planetary Science Letters* **384**, 132-143, doi:10.1016/j.epsl.2013.10.014 (2013).
40. Alibert, C., & McCulloch, M. T. Rare earth element and neodymium isotopic compositions of the banded iron-formations and associated shales from Hamersley, western Australia. *Geochimica et Cosmochimica Acta* **57**, 187-204, doi:10.1016/0016-7037(93)90478-F (1993).
41. Bau, M., Höhndorf, A., Dulski, P., & Beukes, N. J. Sources of rare-earth elements and iron in Paleoproterozoic iron-formations from the Transvaal Supergroup, South Africa: evidence from neodymium isotopes. *The Journal of Geology* **105**, 121-129, doi:10.1086/606152 (1997).
42. Nance, W. B., & Taylor, S. R. Rare earth element patterns and crustal evolution—I. Australian post-Archean sedimentary rocks. *Geochimica et Cosmochimica Acta* **40**, 1539-1551, doi:10.1016/0016-7037(76)90093-4 (1976).
